# Anchor-controlled generative adversarial network for high-fidelity electromagnetic and structurally diverse metasurface design

**DOI:** 10.1515/nanoph-2025-0210

**Published:** 2025-07-15

**Authors:** Yunhui Zeng, Hongkun Cao, Xin Jin

**Affiliations:** Shenzhen International Graduate School, 12442 Tsinghua University, Shenzhen 518055, China; Peng Cheng Laboratory, Shenzhen 518055, China

**Keywords:** metasurface design, generative model, high-fidelity, diverse design

## Abstract

Metasurfaces, capable of manipulating light at subwavelength scales, hold great potential for advancing optoelectronic applications. Generative models, particularly Generative Adversarial Networks (GANs), offer a promising approach for metasurface inverse design by efficiently navigating complex design spaces and capturing underlying data patterns. However, existing generative models struggle to achieve high electromagnetic fidelity and structural diversity. These challenges arise from the lack of explicit electromagnetic constraints during training, which hinders accurate structure-to-electromagnetic mapping, and the absence of mechanisms to handle one-to-many mappings dilemma, resulting in insufficient structural diversity. To address these issues, we propose the Anchor-controlled Generative Adversarial Network (AcGAN), a novel framework that improves both electromagnetic fidelity and structural diversity. To achieve high electromagnetic fidelity, AcGAN proposes the Spectral Overlap Coefficient (SOC) for precise spectral fidelity assessment and develops AnchorNet, which provides real-time physics-guided feedback on electromagnetic performance to refine the structure-to-electromagnetic mapping. To enhance structural diversity, AcGAN incorporates a cluster-guided controller that refines input processing and ensures multilevel spectral integration, guiding the generation process to explore multiple configurations. Empirical analysis shows that AcGAN reduces the Mean Squared Error (MSE) by 73 % compared to current state-of-the-art and significantly expands the design space to generate diverse metasurface architectures that meet precise spectral demands.

## 1 Introduction

Metasurfaces constructed of two-dimensional artificial material structures at subwavelength scales have garnered significant attention for unparalleled ability to manipulate intrinsic properties of light, including spectrum [1], [2], amplitude [3], [4], phase [5], [6], polarization [7], [8], and wavefront [9], [10]. This extraordinary capability arises from the vast design flexibility afforded by the spatial and material configurations of meta-atoms, enabling functionalities far beyond those of natural materials. Leveraging this design flexibility, recent advancements in metasurface design have led to the realization of novel functionalities such as light field imaging [11], holographic display [12], perfect absorption [13], vortex beam generation [14], optical encryption [15], and optical communication [16], showcasing the potential of metasurfaces to revolutionize optical technologies.

Even though modern numerical methods allow for the calculation of the electromagnetic (EM) response of complex structures and diverse materials, the design of metasurfaces is still challenging owing to the nonintuitive and nonunique relationship between physical structures, material properties, and their EM responses [17]. Traditionally, metasurface design relies on physics-inspired methods and human expertise, including insights from analytical models, experience from previous designs, and scientific intuition. Techniques such as resonant phase control [18], propagation phase control [19], and geometric phase control [20], used independently or collectively [7], [21], are pivotal for precise phase response tailoring. However, these methods constrain the design space, limiting innovation primarily to meta-atom configurations, which highlights a related shortcoming: the fundamental theory underpinning metasurfaces is not yet well-established [22]. As design complexity increases, the traditional expert-knowledge–based paradigm becomes less effective [23]. Furthermore, the widely used trial-and-error method, combined with extensive scanning, is constrained by its limited optimization space and the time-consuming process of solving Maxwell’s equations [24].

Deep learning (DL), a subset of artificial intelligence (AI), has emerged as a transformative tool for metasurface design, effectively addressing the challenges posed by traditional methods [25], [26], [27], [28]. By mapping the complex relationships between metasurface parameters and their EM responses, DL facilitates direct design processes while significantly reducing reliance on computationally expensive simulations [29], [30], [31], [32]. Among various DL-driven approaches, Generative Adversarial Networks (GANs) [33], [34], [35], [36] stand out due to their capacity to learn intricate data distributions and generate diverse metasurface structures. This capability not only alleviates the limitations inherent in expert-knowledge–based paradigms – such as their restricted design space and dependence on trial-and-error methodologies – but also paves the way for enhanced design flexibility and innovation in metasurface design. However, GANs often generate outputs that resemble training data without precise control over specific characteristics. To address this, Conditional Generative Adversarial Networks (CGANs) [37], [38], [39], [40] address this limitation by introducing conditional inputs, enabling the generation of designs that can align more closely with predefined EM characteristics [41]. Unlike database retrieval methods that are limited to selecting existing structures, GANs learn the underlying data distribution and sample from a continuous latent space, enabling them to generate novel combinations of metasurface parameters that may not explicitly exist in the original dataset [42], [43]. Nonetheless, GAN-based methods still have two critical challenges to address: i) **Limited Electromagnetic Fidelity**: GAN-based methods typically focus on generating visually accurate structures but often lack explicit constraints to ensure high EM fidelity. This deficiency stems from the absence of direct feedback on EM performance during training, making it difficult for models to learn the complex mapping between metasurfaces and their EM responses. As a result, generated designs may align with the visual characteristics of the dataset but fail to meet the precise EM requirements. ii) **Limited Structural Diversity**: Metasurface design involves a one-to-many mapping dilemma, where multiple structures can produce the same EM response. However, GAN-based methods often generate solutions that resemble the most frequently observed configurations in the training dataset. This limitation arises from the lack of mechanisms that facilitate the exploration of diverse configurations capable of achieving the same EM targets, thus limiting the potential diversity of the generated metasurfaces. The resulting lack of structural diversity critically impairs the adaptability of designs to a range of application requirements and manufacturing constraints. This deficiency may obstruct the identification of optimal structures that could enhance performance or address specific functional demands, ultimately undermining the robustness and applicability of metasurfaces.

**Figure 1:**
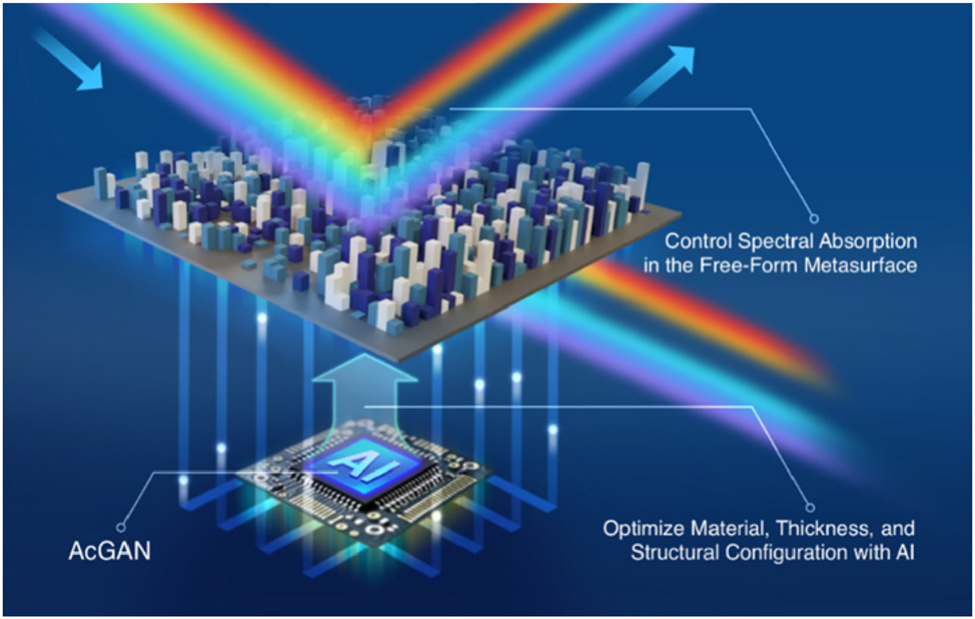
AI-enhanced free-form absorptive metasurface inverse design schematic, illustrating the application of AI to optimize material (indicated by color variations), thickness (represented by height differences), and structural configuration (depicted through column arrangement) to precisely control spectral absorption profiles.

In this study, we focus on the complex task of designing free-form metasurface filters using AI to control and enhance spectral absorption, as demonstrated in Figure 1. To navigate the complex inverse design problem that balances both material and structural properties, we utilize an encoding strategy where key metasurface parameters – including refractive indices, plasma frequencies, and resonator geometries – are mapped into discrete “RGB” channels of color images, capturing a broad design space. To achieve high EM fidelity, our proposed Anchor-controlled Generative Adversarial Network (AcGAN) proposes the Spectral Overlap Coefficient (SOC), a novel metric developed to evaluate the alignment between the generated and target spectral responses, thereby ensuring precise control over the spectral characteristics of the metasurfaces. Furthermore, we develop AnchorNet, a predictive model embedded in the generative framework provides real-time feedback on EM performance during training. This feedback mechanism significantly improves the model’s ability to optimize the complex structure-to-EM mapping. For enhancing structural diversity, AcGAN proposes a cluster-guided controller that promotes the exploration of multiple valid configurations for any given spectral target, effectively addressing the one-to-many mapping dilemma inherent in metasurface design. Combined with our dynamic loss function, this approach shifts the focus from initial data-driven learning to a more balanced optimization of both spectral fidelity and structural diversity. These collective advancements empower AcGAN to not only bridge the gap between visual resemblance and functional EM performance but also establish a robust framework for designing metasurfaces that meet stringent requirements for high EM fidelity and structural diversity in advanced optoelectronic applications. Empirical analysis demonstrates that AcGAN significantly reduces the Mean Squared Error (MSE) by 73 % compared to current state-of-the-art GAN methods and markedly expands the design space to generate diverse metasurface architectures that meet precise spectral demands.

## 2 Methods

**Figure 2:**
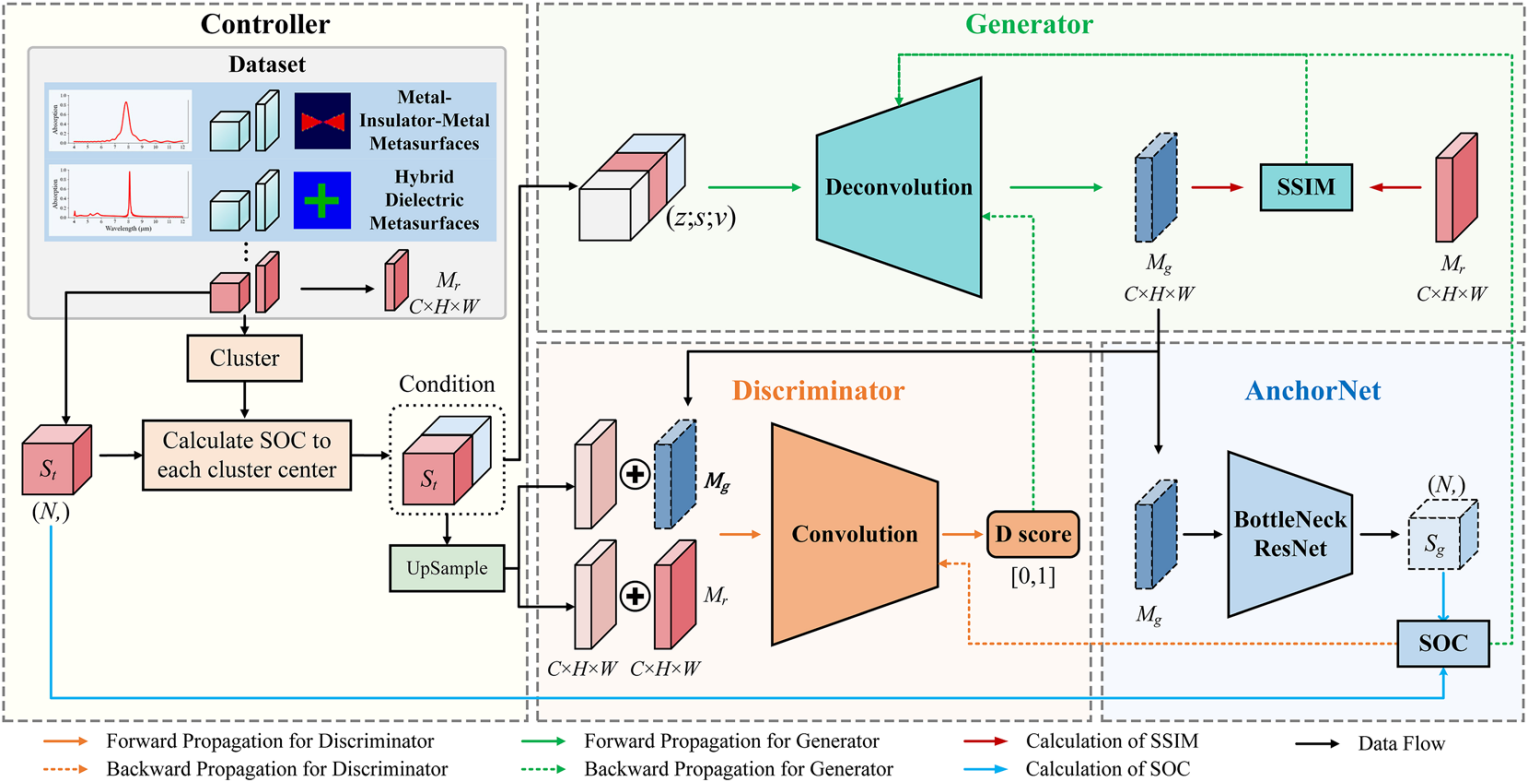
The architecture of AcGAN for metasurface design. The controller manages data clustering and processing, enhancing the structural diversity of designs by enabling the generator to explore various configurations. The generator creates metasurface designs based on these organized data inputs. The discriminator assesses the designs for authenticity and spectral fidelity, ensuring they meet predefined performance standards. AnchorNet guides both the generator and discriminator, providing real-time feedback to improve electromagnetic fidelity.

In this study, we address the complex task of designing free-form metasurface by proposing an advanced AI-driven framework named AcGAN, aiming to enhance both EM fidelity and structural diversity in metasurface designs. Figure 2 outlines the AcGAN architecture, which includes four essential components: controller, generator, discriminator, and AnchorNet, each uniquely contributing to enhance EM fidelity and structural diversity of the generated metasurfaces. The process starts with the pretrained AnchorNet predicting spectral properties, laying the groundwork for adversarial training. Initially, the discriminator is calibrated using precomputed control vectors that replace raw spectral data with structured inputs, streamlining the evaluation process. These inputs allow the discriminator to accurately assess the authenticity and spectral fidelity of the designs, ensuring they align with predefined criteria. Training then shifts focus to the generator, which is optimized through a specialized adversarial loss function to enhance its ability to produce structurally diverse and realistic metasurface designs. The training is iterative, with the generator and discriminator being refined alternately to ensure the designs meet the targeted spectral characteristics effectively. Detailed pseudocode is provided in Supporting information S1.

Building on the AcGAN framework, our approach further explores the engineering of metasurface parameters within a defined design space $M\in R^{d}$ to align the spectral response $S\in R^{m}$ of the metasurface with specified target spectra. Employing EM simulation tools such as Lumerical FDTD for forward mapping *F* : *M* → *S*, we obtain reliable predictions of spectral responses for given metasurface. The crux of the inverse design problem is to determine an optimal design parameters m* that minimizes the discrepancy between the spectrum *s*_*g*_ of generated metasurface and the desired target spectrum *s*_*t*_, represented by the optimization problem:

(1) $$m^{*}=\underset{m\in M}{\mathrm{arg min}}\text{Loss}\left(s_{t},s_{g}\right)$$

where $s=F\left(m\right)$ , and Loss quantifies the distance between *s*_*t*_ and *s*_*g*_. To ensure high electromagnetic fidelity in metasurface designs, we propose a novel spectral similarity metric named Spectral Overlap Coefficient (SOC), defined as follows:

(2) $$SOC=1-\frac{\sum \min\left(s_{t},s_{g}\right)}{\sum \max\left(s_{t},s_{g}\right)}$$

where $\min\left(s_{t},s_{g}\right)$ and $\max\left(s_{t},s_{g}\right)$ are computed element-wise across the spectral vectors *s*_*t*_ and *s*_*g*_. This formula quantitatively measures the extent of spectral overlap, providing a direct assessment of similarity that is especially useful for complex spectral features such as resonance peaks and specific absorption bands. An SOC value nearing zero signifies high similarity, which offers a direct and adaptable measure for spectral congruence, making it invaluable for evaluating and optimizing metasurface designs across diverse spectroscopic applications. SOC directly quantifies the extent of spectral overlap, offering a more granular and accurate measure of spectral congruence. This is particularly advantageous as it ensures a comprehensive alignment of all spectral features, critically assessing the match between peaks and troughs within the spectra. The adoption of SOC transforms our ability to design metasurfaces with high precision, aligning closely with specified EM requirements and surpassing the limitations of conventional design methodologies, as detailed in Supporting information S2. Additionally, the framework seeks to maximize the differences both between *M*_*t*_ and *M*_*g*_, as well as among multiple *M*_*g*_ configurations, thereby promoting greater diversity in the metasurface designs.

**Figure 3:**
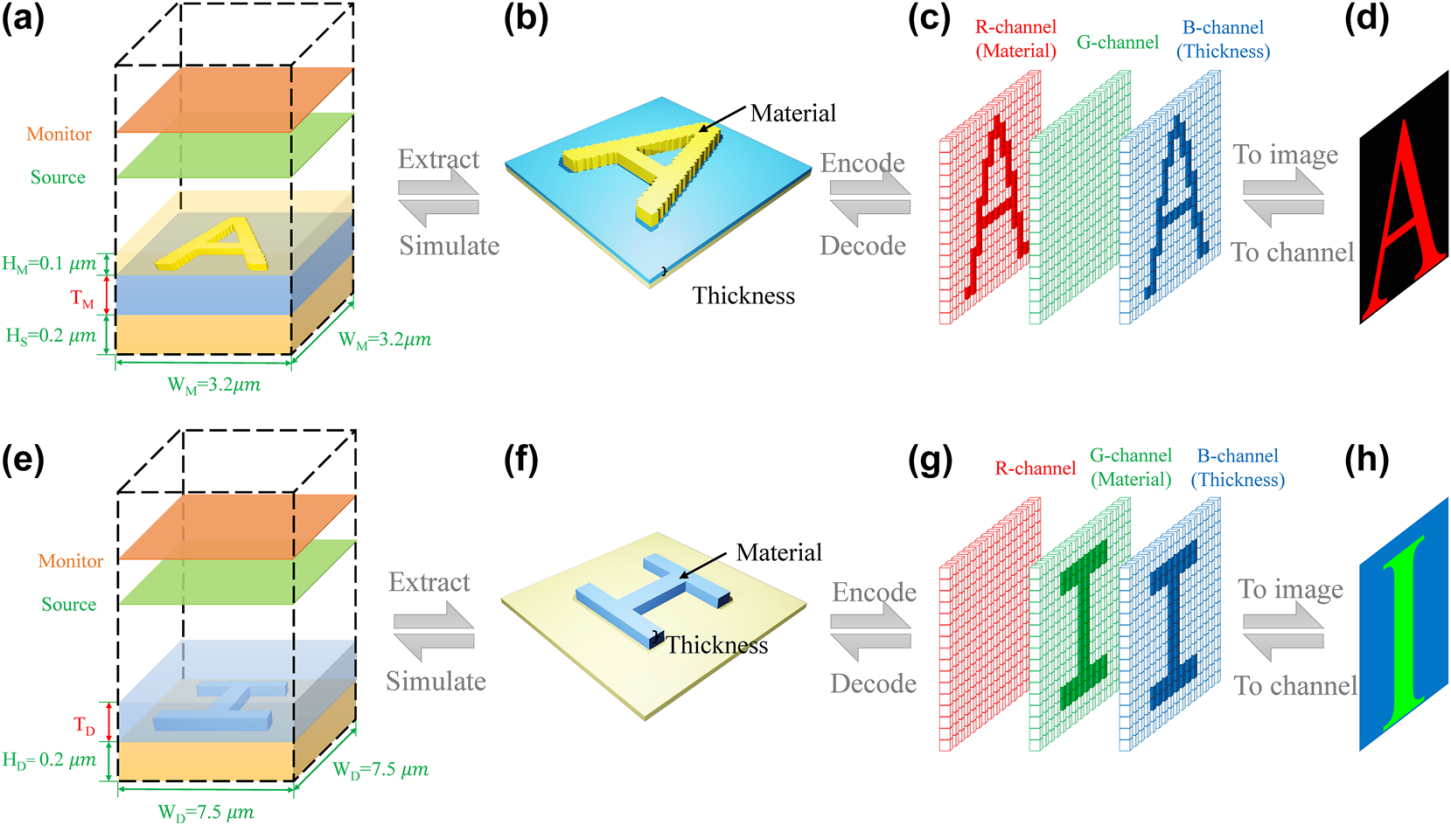
Schematic diagram of coding and decoding process of AcGAN. (a) and (e), the MIM structure (3.2 × 3.2 μm^2^ unit cell) includes a 0.2 μm metal layer, a variable-height Al_2_O_3_ dielectric layer, and a freeform resonator of 0.1 μm height, while the hybrid structure (7.5 × 7.5 μm^2^ unit cell) features a 0.2 μm metal layer with a dielectric freeform resonator of unspecified height. (b) and (f) show the 3D-rendered metasurfaces: the MIM resonators are composed of gold, silver, or aluminum with plasma frequencies of 1.91 PHz, 2.32 PHz, or 3.57 PHz, and dielectric layers of 100, 200, or 300 nm thickness; the hybrid resonators are made of ZnSe, Si, or Ge with refractive indices of 2.41, 3.42, or 4.01, and dielectric layers of 500, 750, or 950 nm thickness. In (c) and (g), the structures are encoded into 64 × 64 × 3 RGB images. For MIM structures, the red channel encodes both the metal material properties and resonator geometry, with the green channel set to zero. For hybrid dielectric structures, the green channel encodes both the dielectric material properties and geometry, with the red channel set to zero. In both cases, the blue channel encodes the thickness of the dielectric layer. (d) and (h) present the final decoded images representing the letters “A” (MIM) and “I” (Hybrid).

In our inverse design framework, two types of metasurfaces are scrutinized, metal–insulator–metal (MIM) constructs characterized by broad Lorentzian absorption spectra arising from plasmonic resonances at the metal–dielectric interface [44]. These spectra are pivotal for applications demanding robust thermal emissivity and efficient photothermal energy conversion. Hybrid dielectric metasurfaces, wherein subwavelength cavity resonances yield Fano-resonant profiles, offering sharply defined spectral features optimal for discerning optical sensor technologies [45]. This bifurcation presents a complex challenge, necessitating a modeling approach capable of accommodating the significant spectral divergences inherent to each metasurface type. We encode two types of metasurfaces into standardized RGB images with dimensions of C × H × W, where C is the number of color channels, and H and W are the image height and width, respectively. For **MIM structures**, the red channel encodes the plasma frequency (*ω*_*p*_) of the metallic resonators, the blue channel records the dielectric layer thickness (*d*, in nanometers), and the green channel is set to zero. For **hybrid dielectric structures**, the green channel encodes the dielectric refractive index (*n*), the blue channel again records the dielectric thickness (*d*), and the red channel is set to zero. In addition, the spectral response ranging from 4 to 12 μm is uniformly discretized into *N* points. This encoding strategy standardizes diverse metasurface configurations into a unified RGB representation, facilitating the training of AcGAN and enabling systematic learning across both metasurface types, as illustrated in Figure 3.

Our framework employs an advanced controller mechanism to address the lack of structural diversity, which acts as an intelligent hub within our architecture, orchestrating the flow and preprocessing of spectral data through sophisticated clustering strategies. Specifically, we utilize the K-means clustering algorithm to segment the training spectral dataset $S=\left\{s_{1},s_{2},\ldots ,s_{n}\right\}$ where each *s*_*i*_ represents a unique spectral data point in $R^{m}$ . The algorithm partitions *S* into *k* distinct clusters, optimizing the following objective:

(3) $$\underset{C}{\min}\overset{k}{\underset{i=1}{\sum }}\underset{s\in C_{i}}{\sum }\|s-c_{i}{\|}^{2}$$

where $C=\left\{C_{1},C_{2},\ldots ,C_{k}\right\}$ represents the set of clusters, and *c*_*i*_ is the centroid or cluster center of *C*_*i*_, embodying the average spectral profile of the cluster. These centroids are then used as reference points to compute the SOC for given spectral input, resulting in a *k*-dimensional vector $v=\left[\text{SOC}\left(s,c_{1}\right),\text{SOC}\left(s,c_{2}\right),\ldots ,\text{SOC}\left(s,c_{k}\right)\right]$ . This vector quantitatively describes the input’s alignment with preidentified spectral categories, enriching the input representation with both detailed and contextual spectral information. The resultant vector *v* is then concatenated with the original spectral data *s* to form a comprehensive control vector $u=\left[s;v\right]$ , which is then input into the generator and discriminator. This enriched input empowers the generator to explore a wider design space, promoting the creation of diverse and functionally tailored metasurfaces. By integrating both detailed and aggregated spectral data, the controller ensures designs not only vary more broadly but also align closely with desired spectra, addressing the challenge of structural diversity problems.

**Figure 4:**
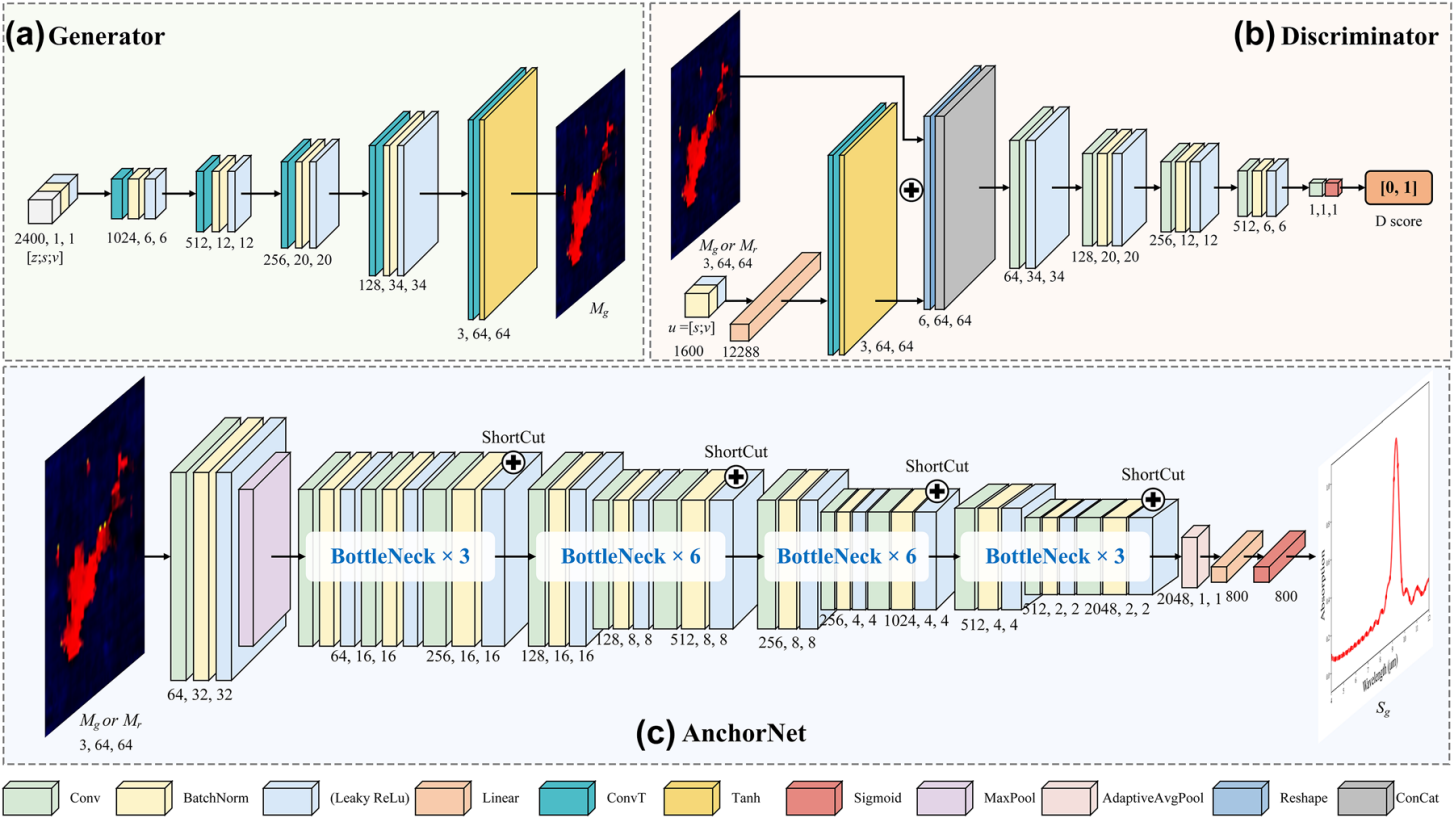
Detailed architectural overview of AcGAN components. (a) Generator: transforms control vectors *u* into metasurface structures *M*_*g*_ using deconvolutions; (b) Discriminator: assesses metasurface designs by applying convolutions to predict authenticity scores ranging from 0 to 1; (c) AnchorNet: predicts spectral responses *s*_*g*_ from metasurface by leveraging bottleneck layers.

Armed with the comprehensive control vector *u* – rich in both granularity and contextual insight – the generator is poised to harness these data for metasurface design. As depicted in Figure 4(a), the generator, equipped with a control vector *u* and a latent vector $z∼N\left(0,1\right)$ (800-dimensional), uses deconvolutional layers to map enriched spectral inputs into spatial structures. The latent vector introduces stochasticity, enabling the model to generate diverse structural solutions for a given spectral target. These layers, coupled with a noise vector for randomness, facilitate a broad exploration of design spaces, which is crucial for achieving one-to-many mappings in metasurface designs. The process is refined through up-sampling, which ensures the preservation of essential spectral features, allowing the generator to produce diverse and functionally effective metasurfaces. The generator’s effectiveness is quantified by a loss function *L*_*G*_, comprising three pivotal components:

(4) $$L_{G}=\gamma L_{\mathrm{adv}}^{G}+\alpha L_{\mathrm{spectral}}+\beta L_{\mathrm{structural}}$$

The *α* and *β* represent the weights for the spectral and structural losses, respectively, and *γ* denotes the weight for the adversarial losses. The adversarial loss component is defined as: $L_{\mathrm{adv}}^{G}=-E_{z∼p_{Z}\left(z\right),u∼p_{U}\left(u\right)}\left[\text{log}D\left(G\left(z,u\right),u\right)\right]$ where $E_{z∼p_{Z}\left(z\right),u∼p_{U}\left(u\right)}$ denotes the expectation over the distributions of latent vectors *z* and control vectors *u*. Here, $G\left(z,u\right)$ is the generator’s output for a given latent vector *z* and control vectors *u*, where $D\left(G\left(z,u\right)\right)$ represents the discriminator’s assessment of how real or fake the generated metasurface. $L_{\mathrm{spectral}}=\text{SOC}\left(s_{g},s_{t}\right)$ measures the spectral similarity, ensuring the generated metasurface aligns with the target spectrum. The structural loss *L*_structural_ is calculated using the Structural Similarity Index (SSIM) [46] between the generated and referenced samples: $L_{\mathrm{structural}}=\text{SSIM}\left(M_{g},M_{r}\right)$ . This metric helps regularize the similarity between generated metasurfaces and reference structures, encouraging that different regions of the latent space z correspond to distinct metasurface designs. Parameters *α* and *β* strategically balance the spectral and structural loss components within the loss function, tailoring the generator’s output to meet specific operational demands while promoting structural diversity. This strategy guarantees that the generated designs not only effectively deceive the discriminator, demonstrating their realistic characteristics, but also accurately meet the targeted EM specifications and display significant structural diversity, thereby increasing their practical utility across various applications.

The discriminator, as depicted in Figure 4(b) is integral to the AcGAN framework, tasked primarily with validating the authenticity of the metasurface designs generated by the generator. It employs a sophisticated convolutional network to critically assess if the generated designs accurately reflect real metasurface EM properties. The discriminator assesses the quality of the synthesized designs using both learned features and heuristics from the training phase. It acts as the critical feedback component, thus guiding the generative process toward the production of metasurfaces with enhanced practical applicability. The discriminator’s functionality is meticulously evaluated through a composite loss function *L*_*D*_:

(5) $$L_{D}=\gamma \left(L_{\mathrm{adv}}^{D}+L_{\mathrm{mismatch}}\right)+\alpha L_{\mathrm{spectral}}$$

where the adversarial loss is expressed as: $L_{\mathrm{adv}}^{D}=E_{\left(M_{r},u\right)∼p_{\mathrm{data}}\left(M_{r},u\right)}\left[\text{log}D\left(M_{r},u\right)\right]-E_{z∼{\text{p}}_{Z}\left(z\right),u∼p_{U}\left(u\right)}\left[\text{log}\left(1-D\left(G\left(z,u\right),u\right)\right)\right]$ . This component compares the discriminator’s predictions for referenced metasurface data $\left(M_{r},u\right)$ , sampled from the dataset, and generated data $G\left(z,u\right)$ from the generator. The first term, $E_{\left(M_{r},u\right)∼p_{\mathrm{data}}\left(M_{r},u\right)}\left[\text{log}D\left(M_{r},u\right)\right]$ , encourages the discriminator to correctly identify real metasurfaces, maximizing the log probability of recognizing real designs. The second term, $E_{\left(z∼p_{Z}\left(z\right),u∼p_{U}\left(u\right)\right)}\left[\text{log}\left(1-D\left(G\left(z,u\right),u\right)\right)\right]$ , penalizes the discriminator for falsely classifying generated metasurfaces as real, pushing it to distinguish between authentic and generated samples effectively. The mismatch loss *L*_mismatch_ enhances the discriminator’s ability to detect inconsistencies between the referenced and generated metasurface, which is computed as:

(6) $$L_{\mathrm{mismatch}}=-E_{z∼p_{Z}\left(z\right),u^{\prime }∼p_{U}\left(u^{\prime }\right)}\left[\log\left(1-D\left(G\left(z,u^{\prime }\right),u\right)\right)\right]$$

where *u*′ represents a control vector that mismatches the intended input conditions for *z*, encouraging the discriminator to penalize incorrect spectral-structure pairings. This loss is inspired by widely adopted practices in conditional GANs [47], [48], [49], where mismatch regularization improves conditional consistency. In our case, it encourages the generated metasurfaces to precisely match the given spectral targets, which is essential for reliable metasurface inverse design. The *L*_spectral_ here refers to the same spectral loss term defined in Eq. (4).

Building upon the intricate interplay between the generator and discriminator within our AcGAN framework, AnchorNet emerges as a pivotal advancement, depicted in Figure 4(c). It incorporates a tailored Bottleneck ResNet [50] architecture, where residual shortcuts deliver structural features from earlier layers directly to deeper ones, helping preserve geometric detail and improve the accuracy of spectral-response prediction. AnchorNet is finely tuned to minimize $\text{SOC}\left(\hat{s},s\right)$ , where $\hat{s}$ is the predicted spectrum, and *s* is the ground truth spectrum obtained from EM simulations. An early stopping mechanism is integrated into the training protocol to halt the learning process after a predetermined number of epochs without improvement in training loss, ensuring computational efficiency and preventing overfitting. Integral to the AcGAN architecture, AnchorNet serves as a fast and reliable surrogate model specifically tailored to predict spectrally resolved responses of complex MIM and hybrid metasurfaces [51], [52], enabling efficient evaluation during training. Specifically developed to assess the EM performance of structures generated by AcGAN, AnchorNet surpasses the conventional visual assessment criteria employed in CGANs. AnchorNet is pretrained and its parameters are frozen during GAN training, following a decoupled scheme to enhance training stability and efficiency [52]. It focuses on aligning EM responses, enabling the optimization of metasurfaces for high EM fidelity, independent of visual resemblance to referenced designs.

The AcGAN framework integrates a carefully designed loss function that is crucial for balancing spectral precision and structural diversity in metasurface design. This loss function governs the interaction between the generator and discriminator to ensure that each design meets stringent spectral standards while exhibiting significant structural variation:

(7) $$\begin{array}{cc} L & =\underset{G}{\min}\underset{D}{max}L\left(D,G\right)\\ & =\gamma \left(L_{\mathrm{adv}}+L_{\mathrm{mismatch}}\right)+\alpha L_{\mathrm{spectral}}+\beta L_{\mathrm{structural}}\\ & =\gamma \left(\begin{array}{c} E_{\left(M_{r},u\right)∼p_{\mathrm{data}}\left(M_{r},u\right),u∼p_{U}\left(u\right)}\left[\log D\left(M_{r},u\right)\right]\\ +E_{Z∼p_{Z}\left(z\right),u∼p_{U}\left(u\right)}\left[\log\left(1-D\left(G\left(z,u\right),u\right)\right)\right]\\ -E_{z∼p_{Z}\left(z\right),u^{\prime }∼p_{U}\left(u^{\prime }\right)}\left[\log\left(1-D\left(G\left(z,u^{\prime }\right),u\right)\right)\right] \end{array}\right)\\ & \;+\alpha \text{SOC}\left(s_{g},s_{t}\right)+\beta \text{SSIM}\left(M_{g},M_{r}\right) \end{array}$$

where adversarial loss *L*_adv_ enables the generator to improve in deceiving the discriminator by making generated metasurfaces more realistic, the mismatch loss *L*_mismatch_ ensures that the generated metasurface aligns with the control vectors, further enhancing design accuracy, the spectral loss ensures that the generated metasurfaces’ spectral responses match the target spectra, and the structural loss encourages structural diversity by comparing the generated metasurfaces to referenced designs. This formulation ensures that each generated design meets stringent spectral criteria while achieving structural diversity. AGAN generates key parameters such as *ω*_*p*_, *d* and *n*, facilitating the exploration of new materials and structures.

## 3 Results

To validate the innovative contributions of our AcGAN framework, we conducted a series of experiments focusing on key performance metrics such as spectral fidelity, structural diversity, and computational efficiency. Specifically, we tested AcGAN’s ability to generate metasurface designs that meet precise spectral response criteria while overcoming the limitations of existing methods in structural diversity. By employing the novel SOC alongside traditional MSE, we quantitatively assessed the accuracy of the generated designs. Additionally, we explored AcGAN’s capacity to generate diverse metasurface configurations for the same target spectrum, leveraging its one-time training advantage for rapid and efficient design iterations. All the experiments were conducted on a computational setup of Intel Xeon E5-2680 CPU (2.50 GHz) and an NVIDIA GeForce RTX 3090 GPU with 24 GB of VRAM, operating Python 3.9.12 on the Ubuntu Linux platform.

**Figure 5:**
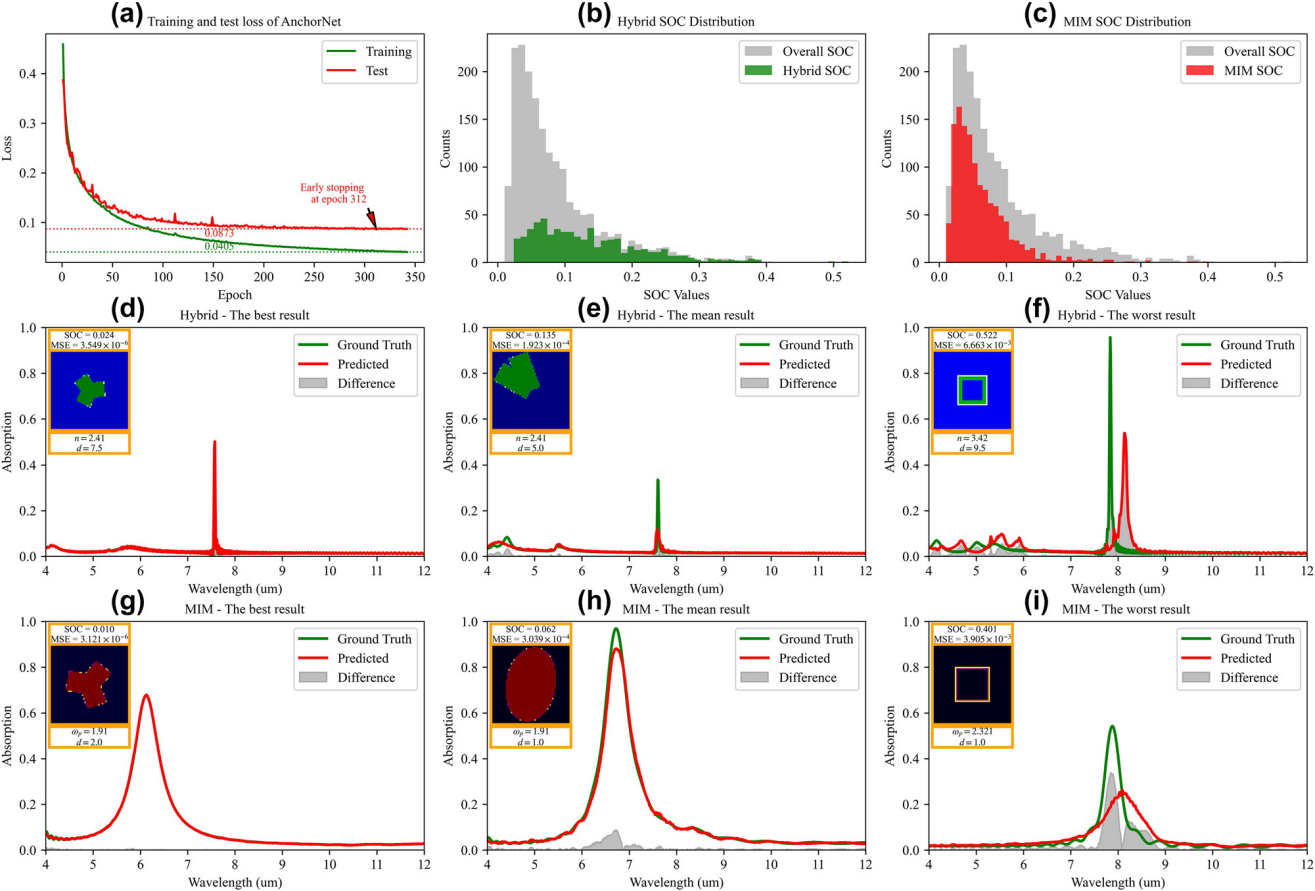
Performance evaluation and spectral predictions of AnchorNet. (a) Training and validation SOC loss curves with early stopping implementation. (b)–(c) SOC distributions: SOC distributions for hybrid (b) and MIM (c) metasurfaces indicating predictive accuracy. (d)–(i) Spectral comparisons for hybrid and MIM structures: displays best, mean, and worst result for hybrid (d)–(f) and MIM (g)–(i) metasurfaces, with insets showing respective metasurface. Red and green lines represent actual and predicted spectra of AnchorNet, respectively. Here, *ω*_*p*_ denotes the plasma frequency of the metal (for MIM structures), *n* is the refractive index of the dielectric resonator (for hybrid structures), and *d* represents the dielectric layer thickness (for both structures).

We firstly evaluated AnchorNet’s performance within the AcGAN framework using a dataset of 18,768 metasurface structures, with one-third categorized as hybrid and two-thirds as MIM. To ensure thorough testing, 90 % of the dataset was allocated for training, and the remaining 10 % served as the test set; the detailed hyperparameter setting of AnchorNet is shown in Supporting information S3. As illustrated in Figure 5(a), SOC losses decreased progressively over 342 epochs, with training ceasing upon reaching an early stopping threshold of 30 consecutive epochs without validation loss improvement. The training consumed 136 min, with final SOC losses for training and testing converging to 0.0405 and 0.0807, respectively. After training, AnchorNet could predict the spectrum of metasurface in an average time of 3.6 × 10^−4^ s, reducing computation time to approximately 1/1,600,000th of the 560.48 s required by FDTD simulations, accelerating the evaluation process for metasurface inverse design, enabling faster iterations and enhancements.

The assessment of AnchorNet across the dataset revealed differences between the hybrid and MIM categories, as detailed in Figure 5(b) to (i). The SOC distribution in Figure 5(b) and (c) demonstrates that MIM structures are predominantly concentrated in lower SOC intervals, indicating higher prediction accuracy compared to Hybrid structures. Quantitatively, the average SOC for MIM structures is 0.0622, which is substantially lower than that of Hybrid structures (0.1346). This result reflects that the network yields more accurate spectral predictions for MIM structures, whose responses are typically characterized by smoother Lorentzian profiles. Figure 5(d)–(i) explicitly demonstrate AnchorNet’s spectral prediction capabilities by presenting instances with the minimum, mean (close to the average SOC), and maximum SOC metrics. Specifically, Figure 5(d)–(f) for hybrid structures and Figure 5(g)–(i) for MIM structures illustrate the accuracy of spectral predictions through comparative plots that juxtapose predicted spectra with ground truth spectra. The improved accuracy for MIM structures is due to their simpler Lorentzian profiles, which are smoother and more predictable than the complex, asymmetric Fano resonances of hybrid structures. These Fano resonances, with sharp variations from quantum interference, present significant predictive challenges. Additionally, the spectral comparison in Figure 5(e) and (h) highlights a key discrepancy: despite a higher MSE in Figure 5(h), the spectral alignment closely matches the ground truth, particularly around peak regions. In contrast, Figure 5(e) shows significant deviations at peak intensities and across broader spectral regions, highlighting MSE’s limitations as a reliable metric. SOC, by providing a more accurate measure of spectral similarity, proves superior to MSE in evaluating spectral fidelity. Inspired by Metric Learning [53], we evaluated Euclidean-based metrics (e.g., MSE and MAE) and SOC for spectral data dimensionality reduction. As detailed in Supporting information S4, our analysis using Principal Component Analysis (PCA) [54], Locally Linear Embedding (LLE) [55], t-Distributed Stochastic Neighbor Embedding (t-SNE) [56], and Autoencoders (AE) [57] revealed Euclidean-based metrics’ limitations in distinguishing classes. In contrast, SOC improved class separability, as shown in Figure S2 in Supporting information S4, demonstrating its effectiveness in preserving intrinsic spectral properties.

To evaluate the performance of our AcGAN method against existing metasurface inverse design techniques, Table 1 provides a comparison across key metrics including training and generation efficiency, MSE, and SOC. MSE is computed as the average squared difference between the generated spectral response *s*_*g*_ and the target spectrum *s*_*t*_ across all discretized spectral points:

(8) $$MSE=\frac{1}{N}\overset{N}{\underset{i=1}{\sum }}{\left(s_{g,i}-s_{t,i}\right)}^{2}$$

where *N* is the number of spectral points within the range of 4–12 μm. SOC is defined as:

(9) $$SOC=1-\frac{\overset{N}{\underset{i=1}{\sum }}\min\left(s_{g,i},s_{t,i}\right)}{\overset{N}{\underset{i=1}{\sum }}\max\left(s_{g,i},s_{t,i}\right)}$$

**Table 1:** Comparison of metasurface design methods based on training and design efficiency, MSE, and SOC: “Training time” indicates the time required for model training, while “Generation time” measures the time needed to design a metasurface that matches the target spectrum. MSE and SOC assess the spectral fidelity of the designed metasurface relative to the desired spectrum. “One-time training” indicates whether the model requires retraining for new spectral targets. All the experiments were conducted on a computational setup of Intel Xeon E5-2680 CPU (2.50 GHz) and an NVIDIA GeForce RTX 3090 GPU with 24 GB of VRAM, operating Python 3.9.12 on the Ubuntu Linux platform, with all methods benchmarked under identical hardware and software settings. Bold values denote the best performance for each metric.

Method	Training time	Generation time	MSE	SOC	One-time training
Physics-inspired	–	Days or months	–	–	No
GA [58]	–	3.89 h	2.120 × 10^−2^	0.564	No
PSO [59]	–	2.23 h	2.010 × 10^−2^	0.557	No
DE [60]	–	4.45 h	2.070 × 10^−2^	0.572	No
DNN [61]	13.4 h	6.1 × 10^−4^s	1.500 × 10^−2^	0.471	Yes
VAE [62]	16.8 h	6.4 × 10^−4^s	1.450 × 10^−2^	0.454	Yes
CGAN [35]	9.62 h	4.2 × 10^−4^s	4.151 × 10^−3^	0.274	Yes
**AcGAN**	**4.16 h**	**4.2 × 10** ^**−4**^ **s**	**1.120 × 10** ^**−3**^	**0.139**	**Yes**

Detailed hyperparameter settings and analysis for AcGAN are provided in Supporting information S5. The hyperparameter settings for comparative methods were adopted from their original papers, and all methods were tested on the same dataset. The results are based on a random selection of 100 spectral data points from the test set. For each data point, corresponding metasurface structures were generated and then simulated using Lumerical FDTD to obtain what can be considered the ground truth spectrum of the designed structures. To address the challenge of limited structural diversity and assess the robustness of designs generated by AcGAN, 256 distinct latent vectors were employed for each target spectrum to explore variations in design accuracy and consistency. Thanks to CUDA’s parallel computing capabilities, generating 256 metasurfaces takes nearly the same time as generating a single one. The design with the lowest SOC relative to the target spectrum is selected as the final design. Traditional physics-inspired methods, in contrast, demonstrate significant inefficiencies as they often require days to months to design a single metasurface and do not support one-time training. Heuristic algorithms like GA [58], PSO [59], and DE [60] are faster but achieve only moderate SOC and MSE, indicating suboptimal spectral fidelity. In contrast, AI-based techniques such as DNN [61], VAE [62], and CGAN [35] significantly reduce prediction latency to milliseconds while improving SOC and MSE. Among these, our AcGAN method excels by recording the fastest generation time of only 4.2 × 10^−4^ s, and achieving the lowest MSE (1.120 × 10^−3^) and SOC (0.139). Additionally, the one-time training feature of machine learning methods presents considerable advantages over the iterative, resource-intensive nature of traditional and heuristic approaches.

**Figure 6:**
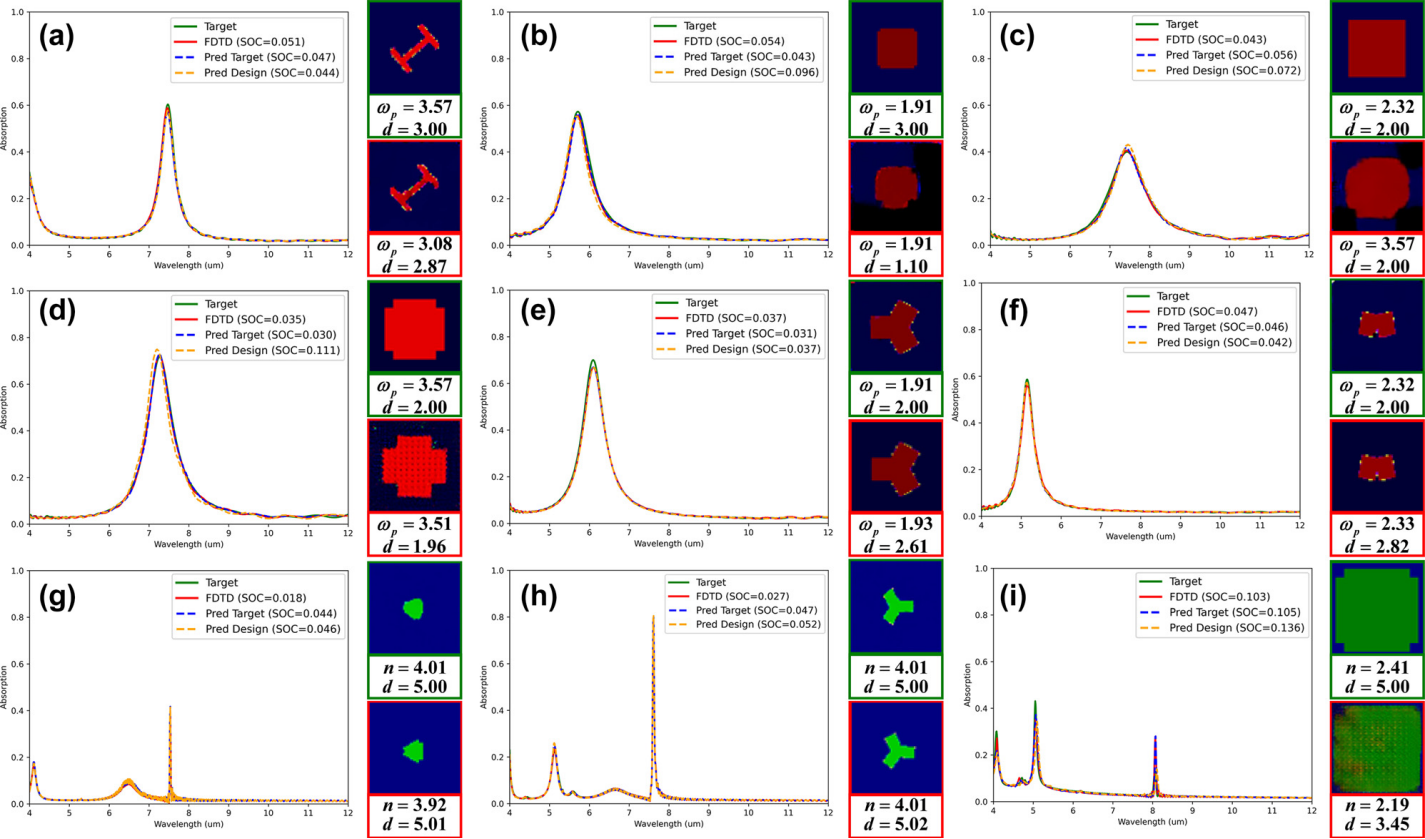
Randomly selected spectra from the test dataset served as input targets for AcGAN. This figure presents a comparative analysis between the target spectra (solid green lines) and the corresponding spectra of metasurfaces designed by AcGAN after FDTD simulation (solid red lines). Dashed blue and yellow lines represent the spectra predicted by AnchorNet for referenced metasurface and AcGAN-designed metasurfaces, respectively. To the right of each plot, green-framed images depict reference metasurface structures with material and thickness parameters, while red-framed images show AcGAN-designed metasurfaces, annotated with material types and thicknesses (*t* in nanometers), and plasma frequency (ω*P*) values are given in PHz.

**Figure 7:**
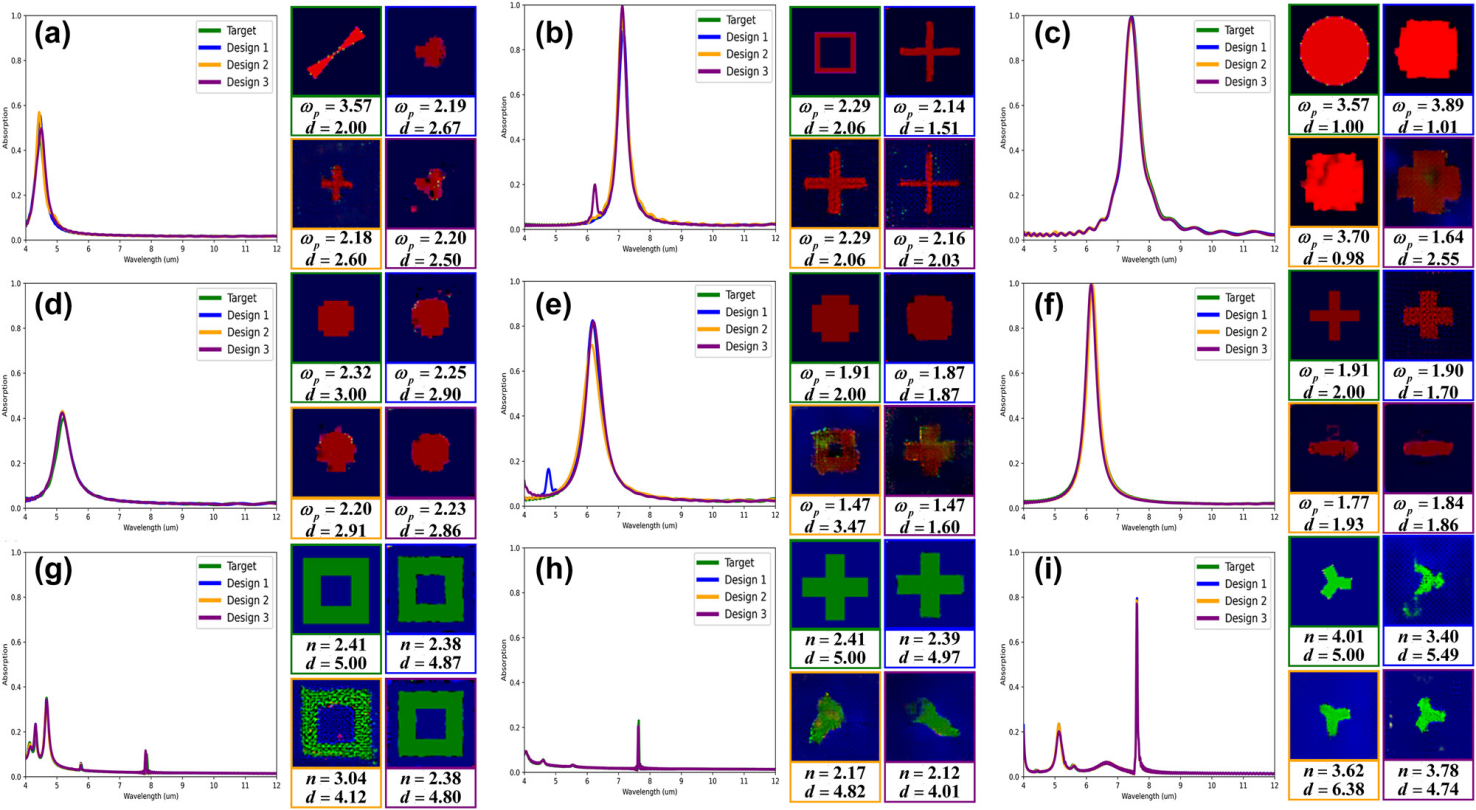
Demonstrating AcGAN’s capability for design diversity. This figure compares target spectra (solid green lines) with three distinct spectra generated by AcGAN (solid blue, yellow, and purple lines), highlighting AcGAN’s ability to create multiple diverse metasurface designs from a single target specification. Adjacent to each spectral plot, images within green frames depict referenced metasurface structures with detailed material and thickness parameters. Corresponding images in blue, yellow, and purple frames showcase the various designs created by AcGAN, each annotated with specific material types, thicknesses, and plasma frequency values.

Figure 6 presents 9 representative cases from the test dataset, each showcasing the design generated by AcGAN with the lowest SOC relative to the target spectrum, highlighting the model’s ability to achieve high spectral fidelity. The spectra from FDTD simulations of metasurfaces designed by AcGAN closely align with the target spectra, underscoring AcGAN’s unprecedented efficiency and accuracy in generating metasurfaces precisely tailored to specific spectral requirements, a significant advancement over traditional methods. Moreover, the close match between the spectra from referenced metasurface structures and the target spectra predicted by AnchorNet emphasizes AnchorNet’s exceptional predictive capability, which is critical for ensuring high electromagnetic fidelity in the design process. Notably, AcGAN accurately designed MIM structures for Lorentzian spectra and hybrid structures for Fano spectra, successfully distinguishing between different physical mechanisms during training without confusion. This demonstrates AcGAN’s innovative capability to effectively differentiate between distinct metasurface types and their corresponding absorption spectra, showcasing its proficiency not only in achieving high design accuracy but also in understanding and applying different physical mechanisms – a significant advancement in metasurface design. AcGAN demonstrates a notable capacity to design metasurfaces that closely match target spectra while significantly diverging in structural dimensions, material properties, and dielectric thickness, as shown in Figures 6 and 7. Analysis of the three metasurfaces with the lowest SOC reveals that, although their absorption spectra closely match the target, their physical configurations vary significantly. For instance, material properties deviate by an average of 12.9 %, and dielectric thicknesses vary by 20.0 %, highlighting the model’s ability to achieve diverse designs beyond traditional visual constraints. In particular, Figure 7(b) highlights that design1’s thickness is reduced by 26.7 % compared to the referenced metasurface, simplifying the manufacturing process. Despite these variations, the average SSIM between the generated and referenced metasurfaces is 0.727, indicating substantial structural differences while maintaining functional integrity, as shown in Figure 7(b) and (c). While variations in the latent vector *z* introduce observable diversity in the generated structures, we did not observe a consistent or interpretable mapping between specific regions of the latent space and distinct metasurface features. This may suggest that *z* primarily serves to introduce structural variability rather than encoding explicit design semantics, which is consistent with previous observations in generative design literature [63], [64]. Due to the stochastic nature of deep learning-based generative methods, slight leakage between mutually exclusive channels may occasionally occur, for example in Figure 7(e). Although the leakage ratio is low (∼0.23 % per structure), the automatic removal of these pixels during decoding into metasurface structures alters the final design. While this does not significantly affect practical implementation, it represents a methodological limitation. To further mitigate such leakage, stronger constraints could be considered in future iterations of the model.

**Figure 8:**
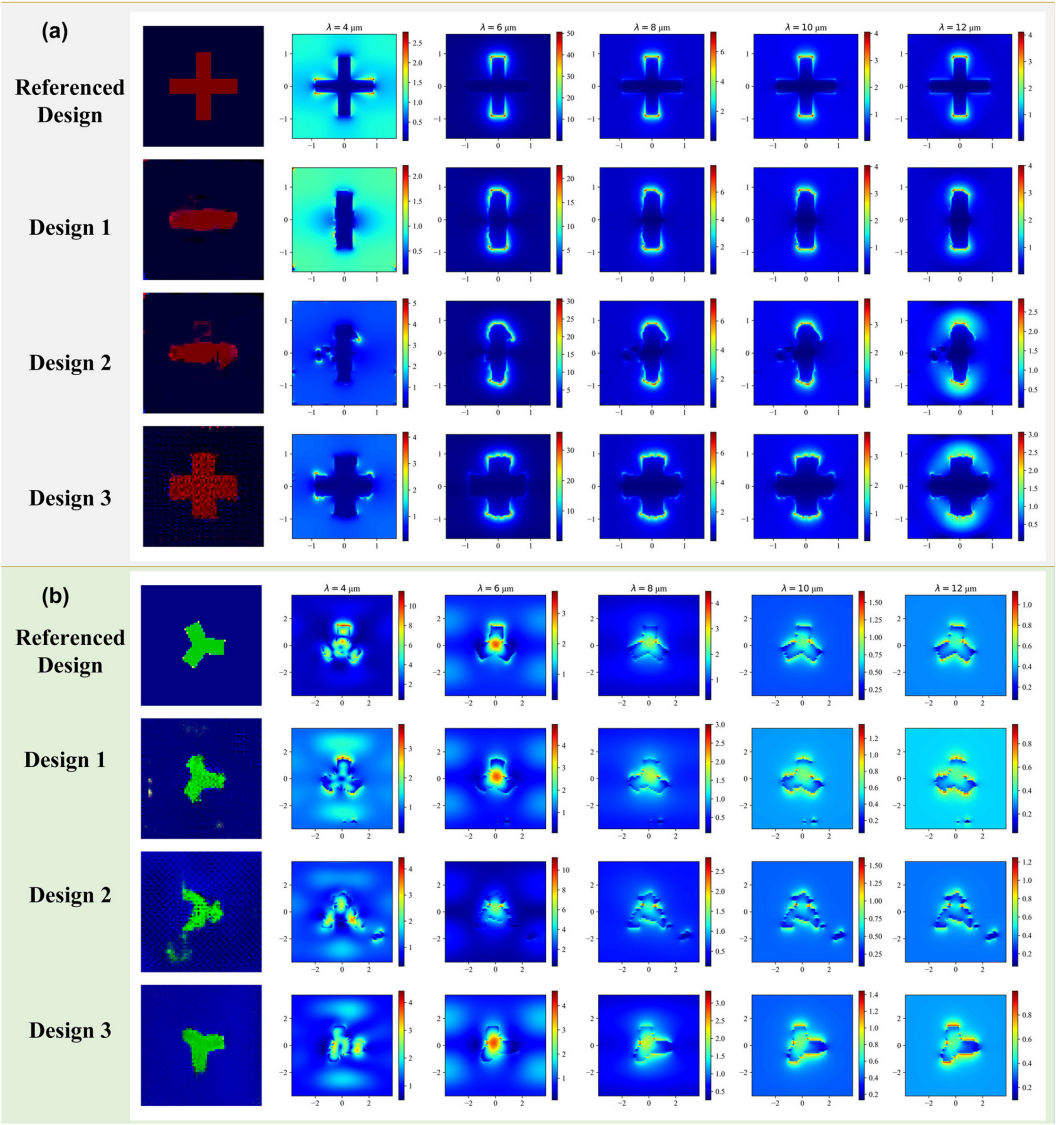
Near-field electric responses in the XY plane for MIM and hybrid metasurface: (a) MIM metasurface: showcases the near-field electric responses at various wavelengths (4 μm–12 μm) for MIM metasurface in Figure 7(f). The depth of the colors i reflects the magnitude of the near-field electric field strength. (b) Hybrid metasurface: presents the near-field electric responses at wavelengths from 4 μm to 12 μm for hybrid metasurface in Figure 7(i).

To further illustrate AcGAN’s ability to enhance design diversity, Figure 8 presents the near-field electric distributions in the XY plane for both MIM and hybrid metasurface structures. This visualization demonstrates AcGAN’s ability to not only match the target spectral responses but also innovate in the spatial arrangement of meta-atoms across various planes. The corresponding electric field variations in the XZ and YZ planes, which exhibit similarly diverse distributions, are discussed in Supporting information S6. The design versatility enabled by AcGAN allows a single imaging system to perform multiple functions, such as standard and polarimetric imaging, without requiring changes to the optical components. This adaptability significantly enhances the visualization of cellular or tissue structures across different depths and orientations. By generating metasurfaces with tailored EM functionalities, AcGAN expands the operational flexibility and efficiency of imaging systems, paving the way for broader applications.

To assess AcGAN’s ability to handle arbitrarily defined spectral challenges, we explored four spectral types: Fano, Lorentzian, Gate, and Gaussian. These spectra were generated according to the details in Supporting information S7, ensuring no same data in the training dataset. For each type, we generated two spectra and simulated 256 metasurface structures per spectrum. The results are shown in Supporting information S8, each panel in Figure S5 contrasts the simulated spectra with the target, highlighting discrepancies in shaded areas and quantifying them with SOC and MSE values. The results show that while AcGAN closely approximates the true spectral characteristics for Fano and Lorentzian resonances Figure S5 a)-d), it exhibits deviations in Gate and Gaussian profiles, particularly at the spectral tails Figure S5 e)-h). This variance suggests AcGAN’s robust performance on spectra present in the database but highlights the need for better model generalization to accommodate theoretically defined but unrepresented spectral types in the training set.

## 4 Discussion

To further analyze the performance of AcGAN for a variety of spectral designs and their relation to the training dataset, we propose the “weighted distance” metric, which is defined as $D_{w}\left(s\right)=\sum_{i=1}^{k}\left(\frac{n_{i}}{N}\cdot \text{SOC}\left(s,c_{i}\right)\right)$ , this metric quantifies the deviation of a spectrum *s* from the cluster centroids $\left\{c_{1},c_{2},\ldots ,c_{k}\right\}$ based on the training data. Here, *n*_*i*_ is the number of spectra in the *i*-th cluster, and *N* is the total number of spectra across all clusters. For empirical analysis, we generated 1,000 spectra for each of the four spectral types and calculated their respective *D*_*w*_. Each spectrum was categorized into intervals of 0.05 in weighted distance, with 10 spectra sampled per interval to ensure uniform coverage. The results are shown in Supporting Information S9, Figure S6 illustrates the correlation between SOC and weighted distance, with ellipses highlighting the general distribution of SOC against weighted distance for each type. Notably, spectra with lower weighted distances typically achieved lower SOC, indicating closer approximation to the target spectral characteristics. This trend was especially pronounced for Lorentzian and Fano spectra. Conversely, the Gate and Gaussian spectra demonstrated lower SOC, underscoring potential challenges in generating these spectra types due to their underrepresentation in the training data. The observed variance in AcGAN’s performance across spectral types highlights the critical importance of training dataset diversity for model generalization. Expanding the dataset to include a broader spectrum of metasurface configurations could enhance the model’s capability to accurately generate designs for a wider array of spectral responses. Future research will focus on augmenting the dataset and refining model algorithms to improve performance across less represented spectra, thereby broadening the practical applications of the AcGAN framework in metasurface design.

To thoroughly evaluate the AcGAN’s robustness and adaptability to varying configurations, we executed a series of ablation studies, the detailed results of which are presented in Supporting information S10. Our ablation analyses underscored the pivotal roles of the cluster controller and AnchorNet’s integration within both the generator and discriminator. The presence of the cluster controller led to a notable decrease in MSE and SOC, indicating higher fidelity EM design. Similarly, enabling AnchorNet in both model components significantly enhanced the fidelity of generated metasurface designs. Adjustments in the adversarial training dynamics, particularly variations in the k-value, defined as the number of generator updates per discriminator update, revealed that a balanced approach is crucial for stable training and optimal model performance. The studies highlighted that a k-value of 2 was optimal, significantly reducing MSE and increasing SOC compared to other configurations. Moreover, the impact of initial spectral loss weighting, represented by the parameter *α*, was profound. Surprisingly, a lower weight (*α* = 0.1) unexpectedly yielded better performance, suggesting that an excessive initial emphasis on spectral fidelity might hinder the model’s ability to generalize across a broader design space. This finding points to the need for a balanced loss function that adequately emphasizes both spectral fidelity and adversarial robustness. Investigations into the effects of latent space dimensions and batch sizes further refined our understanding of model behavior. Optimal latent dimensions and smaller batch sizes tended to improve the model’s precision and stability, indicating that finer granularity in the generation process aids in capturing the nuances of metasurface designs. Collectively, these findings underscore the intricate interdependencies within the AcGAN architecture and highlight the need for careful calibration to maximize performance. These insights are crucial for refining the AcGAN framework to enhance its practical applicability across diverse metasurface design scenarios, thereby extending the capabilities of current computational photonic design methods.

## 5 Conclusions

AcGAN marks a significant advancement in the inverse design of metasurfaces, showcasing an unprecedented combination of high electromagnetic fidelity and extensive structural diversity. This innovative framework, through rigorous empirical testing, has proven to effectively minimize the MSE by 73 % compared to existing GAN approaches, demonstrating its robust capability to meet stringent spectral demands with enhanced precision. Crucially, AcGAN addresses the perennial challenges of electromagnetic fidelity and structural diversity that have impeded prior generative models. By integrating the SOC and AnchorNet, our framework not only assesses but significantly improves spectral fidelity, ensuring that each generated design adheres closely to the desired electromagnetic characteristics. This precision is vital for applications in complex optoelectronic systems, where exact spectral properties are critical for functionality. Furthermore, AcGAN innovatively incorporates a cluster-guided controller, which refines input processing and facilitates the exploration of diverse structural configurations. This feature is essential for overcoming the one-to-many mapping dilemma inherent in metasurface design, allowing for a broader range of functional possibilities within a single design process. The dynamic loss function, shifting focus from data-driven learning to optimizing spectral and structural outcomes, further underscores our method’s adaptability and efficiency.

Looking ahead, future enhancements to AcGAN will focus on several critical areas: 1. **Optimizing AnchorNet**: Enhancing its predictive accuracy, particularly for hybrid structures with complex Fano resonance profiles, which currently present significant challenges. 2. **Enhancing encoding techniques**: Expanding the range of design variables to include more intricate and functional metasurface configurations, thereby broadening the scope of metasurface applications. 3. **Expanding dataset diversity**: Incorporating a broader array of metasurface structures, especially free-form designs, to improve the model’s generalization capabilities and robustness. 4. **Considering manufacturability and fabrication tolerances**: Addressing potential challenges in manufacturing complex metasurface designs by ensuring that the generated structures are not only diverse but also feasible to produce with existing fabrication technologies. This will improve the practical applicability of AcGAN-generated designs in real-world scenarios. In addition, the proposed AcGAN framework can be naturally bridged with classical optimization strategies such as adjoint solvers or evolutionary algorithms [34], [65]. Such hybrid integration holds strong potential for enhancing spectral controllability and improving the robustness of inverse design solutions. Finally, combining AcGAN with classical mode-collapse mitigation strategies [66], [67] – such as architectural innovations [68], diversity-aware regularization [69] – may provide complementary benefits, offering a promising path to further enhance structural diversity.

AcGAN represents not only a methodological breakthrough but also a scalable and robust framework that significantly accelerates the design process in nanophotonic applications. This makes AcGAN a pivotal tool for advancing operational metasurfaces, meeting the evolving demands of optoelectronics and related industries.

## Supplementary Material

Supplementary Material Details
