# Supplementary material for: Anchor-controlled generative adversarial network for high-fidelity electromagnetic and structurally diverse metasurface design
Source: Nanophotonics. 2025 Jul 15;14(17):2923–38. doi: 10.1515/nanoph-2025-0210 (PMC12397733; doi:10.1515/nanoph-2025-0210)
Supplement: Supplementary file 1 — Supplementary Material Details [file j_nanoph-2025-0210_suppl_001.docx]

Supporting information: Anchor-Controlled Generative Adversarial Network for High-Fidelity Electromagnetic and Structurally Diverse Metasurface Design

Yunhui Zeng^1,2^, Hongkun Cao^2*^, Xin Jin^1,2*^,

^1^Shenzhen International Graduate School, Tsinghua University, Shenzhen 518055, China

^2^Peng Cheng Laboratory, Shenzhen 518055, China

***Email:** [jin.xin@sz.tsinghua.edu.cn](mailto:jin.xin@sz.tsinghua.edu.cn) (Xin Jin), [caohk@pcl.ac.cn](mailto:caohk@pcl.ac.cn) (Hongkun Cao)

Section S1. The pseudocode of the training of AcGAN

**Algorithm 1**: AcGAN Training Procedure for Metasurface Design

**Input:** Training pairs $\{\left( M_{r}^{i},s_{t}^{i},u_{i} \right)\}$, where $M_{r}^{i}$ are referenced metasurface, $s_{t}^{i}$ are target spectral samples and $u_{i}$ are control vectors.

**Parameters**: Number of epochs $E$, batch size $B$, learning rate $\eta$, spectral and structural loss weights $\alpha,\beta$, adversarial and mismatch loss weight $\gamma$

1. Initialize parameters $\theta_{G}$ for generator $G$ and parameters $\theta_{D}$ for discriminator $D$ with random weights

2. Pre-train AnchorNet to predict spectral properties from metasurface designs;

3. **For** epoch $=1$ to $E$ **do**

4. Shuffle the training data;

5. Sample minibatch of $m$ noise samples $\{z^{\left( 1 \right)},\ldots,z^{\left( m \right)}\}$ from $p_{Z}\left( z \right)$；sample minibatch of $m$ control vectors $\{u^{\left( 1 \right)},\ldots,u^{\left( m \right)}\}$ from $p_{U}\left( u \right)$；sample minibatch of m examples {$\left( M_{r}^{\left( 1 \right)},s_{t}^{\left( 1 \right)} \right),\ldots,\left( M_{r}^{\left( m \right)},s_{t}^{\left( m \right)} \right)$} from $p_{\text{data}}\left( M_{r},s_{t} \right)$;

6. **For** each batch $\{\left( z,u,x \right)\}$ of size $B$ **do**

//Update the discriminator

7. **For** $k$ steps:

8. $M_{g}\leftarrow G\left( z,u \right)$ //Generate metasurface conditioned on $u$;

9. $D_{\text{real}}\leftarrow D\left( x,u \right)$//Discriminator output for referenced metasurface;

10. $D_{\text{fake}}\leftarrow D\left( M_{g},u \right)$ //Discriminator output generated metasurface;

11. $D_{\text{mismatch}}\leftarrow D\left( M_{g},u' \right)$ //Discriminator output for mismatched data;

12. $L_{D}\leftarrow\gamma\left( L_{adV}^{D}+L_{\text{mismatch}} \right)+\alpha L_{\text{spectral}}$ ; //Calculate the loss for $D$;

13. $\theta_{D}^{'}\leftarrow\theta_{D}-\eta\nabla L_{D}$; //Update $D$ by ascending its stochastic gradient;

14. **End for**

//Update the generator

15. $M_{g}\leftarrow G\left( z,u \right)$ //Generate metasurface conditioned on $u$;

16. $D_{\text{fake}}\leftarrow D\left( M_{g},u \right)$ //Discriminator output generated metasurface;

17. $L_{G}=\gamma L_{adv}^{G}+\alpha L_{\text{spectral}}+\beta L_{\text{structural}}$ ; //Calculate the loss for $G$;

20. $\theta_{D}^{'}\leftarrow\theta_{D}-\eta\nabla L_{D}$; //Update $G$ by ascending its stochastic gradient;

21. **End for**

21. **End for**

**Output:** Trained models D and G.

Section S2. Literature review on spectral similarity evaluation metrics

**Table S1.** Literature review of evaluation matrix of spectral similarity

| Research Work | Year | Journal | Inverse/Forward | matrix |
| --- | --- | --- | --- | --- |
| So S, Rho J.^1^ | 2019 | Nanophotonics | Inverse | MAE |
| Ma W, Cheng F, Xu Y, et al.^2^ | 2019 | Advanced Materials | Inverse | MSE |
| Yeung C, Tsai J M, King B, et al.^3^ | 2020 | ACS Photonics | Forward | MSE |
| Yeung C, Tsai R, Pham B, et al.^4^ | 2021 | Advanced Optical Materials | Inverse | MSE |
| Han X, Fan Z, Liu Z, et al.^5^ | 2021 | InfoMat | Inverse | MSE |
| Yeung C, Tsai J M, King B, et al.^6^ | 2021 | Nanophotonics | Inverse | MSE |
| Mekki-Berrada F, Ren Z, Huang T, et al.^7^ | 2021 | npj Computational Materials | Inverse | MSE |
| Tanriover I, Lee D, Chen W, et al.^8^ | 2022 | ACS Photonics | Inverse | MSE |
| Patel S K, Parmar J, Katkar V.^9^ | 2022 | Renewable Energy | Inverse | MAE |
| Zhang J, Qian C, Fan Z, et al.^10^ | 2022 | Advanced Optical Materials | Inverse | MSE |
| Liu X, Wang P, Xiao C, et al.^11^ | 2023 | Advanced Functional Materials | Inverse | MSE |

In existing works, Mean Squared Error (MSE) and Mean Absolute Error (MAE) are the prevalent metrics for assessing spectral similarity, defined as $MSE\left( s_{t},s_{g} \right)=\frac{1}{n}\sum_{i=1}^{n} \left( s_{t}-s_{g} \right)^{2}$, and $\cdot$ $MAE\left( s_{t},s_{g} \right)=$ $\frac{1}{n}\sum_{i=1}^{n} \left| s_{t}-s_{g} \right|$, respectively. While these metrics are favored in deep learning-based metasurface design due to their straightforward computational properties and robust performance, they often fail to capture critical nuances of metasurface spectral features, such as resonance peaks or specific absorption bands that are crucial yet may constitute only a fraction of the overall spectrum. This oversight can lead to deceptively favorable evaluations, as these minute but essential spectral details are overlooked. To overcome these limitations, our work propose SOC, unlike MSE and MAE, SOC directly quantifies the extent of spectral overlap, offering a more granular and accurate measure of spectral congruence. This metric enhances the assessment of metasurface designs by ensuring that all significant spectral features, especially those critical for specific applications, are accurately matched. The introduction of SOC represents a significant advancement in the field, providing a metric that is not only mathematically sound but also specifically tailored to the unique requirements of metasurface spectral evaluation.

**Reference:**

1. Designing nanophotonic structures using conditional deep convolutional generative adversarial networks. https://www.degruyter.com/document/doi/10.1515/nanoph-2019-0117/html.

2. Ma, W., Cheng, F., Xu, Y., Wen, Q. & Liu, Y. Probabilistic Representation and Inverse Design of Metamaterials Based on a Deep Generative Model with Semi‐Supervised Learning Strategy. *Advanced Materials* **31**, 1901111 (2019).

3. Yeung, C. *et al.* Elucidating the Behavior of Nanophotonic Structures through Explainable Machine Learning Algorithms. *ACS Photonics* **7**, 2309–2318 (2020).

4. Yeung, C. *et al.* Global inverse design across multiple photonic structure classes using generative deep learning. *Advanced Optical Materials* **9**, 2100548 (2021).

5. Han, X., Fan, Z., Liu, Z., Li, C. & Guo, L. J. Inverse design of metasurface optical filters using deep neural network with high degrees of freedom. *InfoMat* **3**, 432–442 (2021).

6. Yeung, C. *et al.* Multiplexed supercell metasurface design and optimization with tandem residual networks. *Nanophotonics* **10**, 1133–1143 (2021).

7. Mekki-Berrada, F. *et al.* Two-step machine learning enables optimized nanoparticle synthesis. *npj Comput Mater* **7**, 55 (2021).

8. Tanriover, I., Lee, D., Chen, W. & Aydin, K. Deep Generative Modeling and Inverse Design of Manufacturable Free-Form Dielectric Metasurfaces. *ACS Photonics* (2022) doi:10.1021/acsphotonics.2c01006.

9. Patel, S. K., Parmar, J. & Katkar, V. Graphene-based multilayer metasurface solar absorber with parameter optimization and behavior prediction using Long Short-Term Memory model. *Renewable Energy* **191**, 47–58 (2022).

10. Zhang, J. *et al.* Heterogeneous Transfer‐Learning‐Enabled Diverse Metasurface Design. *Advanced Optical Materials* **10**, 2200748 (2022).

11. Liu, X. *et al.* Compatible Stealth Metasurface for Laser and Infrared with Radiative Thermal Engineering Enabled by Machine Learning. *Adv Funct Materials* **33**, 2212068 (2023).

Section S3. The detailed hyperparameter setting of AnchorNet

**Table S2.** The detailed hyperparameter setting of AnchorNet

| **Hyperparameter** | **Value** |
| --- | --- |
| $C\times H\times W$ | $3\times64\times64$ |
| N | 800 |
| Learning rate | 0.001 |
| Batch size | 64 |
| Maximum training epochs | 500 |
| Loss function | SOC |
| Early stopping patience | 30 |
| Optimizer | Adam |
| Betas | (0.9, 0.999) |
| Epsilon | $1\times{10}^{-8}$ |
| Valid step | 1 |

Training was initially capped at 500 epochs. However, training was stopped early at 312 epochs due to no improvement in the validation set over 30 consecutive epochs, as measured by the SOC) This decision was based on our empirical determination of hyperparameters, including a learning rate of 0.001, a batch size of 64, and the Adam optimizer with beta values of (0.9, 0.999). These settings align with standard practices in deep learning to optimize training efficiency and effectiveness.

**Section S4.** **Evaluating the impact of spectral similarity evaluation metrics on spectral data dimensionality reduction**


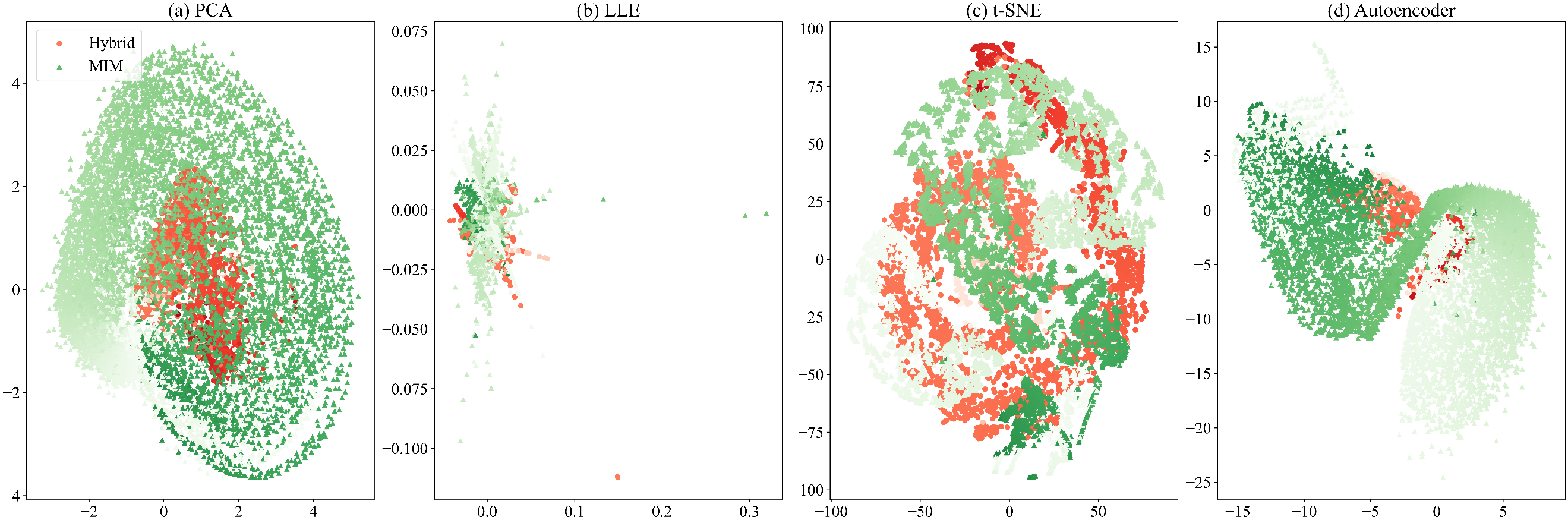


**Figure S1**. Performance of dimensionality reduction techniques: This figure shows the embeddings produced by four methods—(a) PCA, (b) LLE, (c) t-SNE, and (d) Autoencoder—for the Hybrid (red circles) and MIM (green triangles) spectral classes. PCA, LLE, and t-SNE are constructed using Euclidean-distance criteria, while the Autoencoder is trained with mean-squared error (MSE). These plots illustrate the limitations of Euclidean-based similarity metrics in effectively distinguishing between the two spectral classes.





**Figure S2**. Improved spectral data dimensionality reduction with SOC using Autoencoders: (a), (b), and (c) compare the effect of using different loss functions—MSE, SOC, and MAE—in an autoencoder for spectral data dimensionality reduction. (a) shows the results using MSE, where significant overlap occurs between Hybrid (red circles) and MIM (green triangles) spectra. (b) illustrates the results using SOC, highlighting a clear separation between Hybrid and MIM spectra, confirming SOC's effectiveness in improving spectral class differentiation and preserving intrinsic spectral properties. (c) shows the results using MAE, which, similar to MSE.

In this study, we focus on exploring the effects of different loss functions on the dimensionality reduction and classification of spectral data, with a particular emphasis on the comparative performance between traditional Euclidean-distance criteria and SOC. Our experiments initially employed four mainstream dimensionality reduction techniques: Principal Component Analysis (PCA), Locally Linear Embedding (LLE), t-Distributed Stochastic Neighbor Embedding (t-SNE), and Autoencoders (AE), PCA, LLE, and t-SNE are constructed using Euclidean-distance criteria, while the Autoencoder is trained with MSE. The results demonstrated that regardless of the dimensionality reduction method used, the two spectral classes (Hybrid and MIM) remained indistinguishable with Euclidean-distance criteria, highlighting its limitations in preserving spectral characteristics.

To further validate SOC's efficacy in dimensionality reduction and classification, we conducted additional experiments with an autoencoder to compare the effects of MAE, MSE and SOC as loss functions. As illustrated in **Figure S2**, when SOC was employed as the loss function, there was a clear separation and formation of distinct clusters for the Hybrid and MIM data in the reduced dimension space. This demonstrated SOC’s effectiveness in distinguishing spectral responses and revealing intrinsic data features. SOC also preserved physical characteristics, aiding generalization for resonant responses with slight wavelength variations—key for applications like laser cavity design and photonic crystal optimization. Overall, SOC provides a valuable approach for analyzing spectral data and enhancing optical device performance in nanophotonics and optical design.

Section S5. The detailed hyperparameter setting of AcGAN

**Table S3.** The detailed hyperparameter setting of AcGAN

| **Hyperparameter** | **Value** |
| --- | --- |
| Learning rate | 0.001 |
| Batch size | 256 |
| Training epochs | 1000 |
| Loss function | SOC |
| Optimizer | Adam |
| Betas | (0.9, 0.999) |
| Epsilon | $1\times{10}^{-8}$ |
| Latent vector length | 800 |
| $k$ | 800 |
| $\xi$ | 2 |
| $\alpha,$ | 0.1 |
| $\beta$ | 0.1 |
| $\gamma$ | 0.9 |

The AcGAN was rigorously trained to optimize metasurface designs. Training was conducted using the Adam optimizer with a learning rate of 0.001 over 1000 epochs and a batch size of 256. The SOC, assessing the precision in replicating target spectral features, served as the core metric for model evaluation and optimization. Initially, the spectral and structural loss weights $(\alpha,\beta)$ were set at 0.1, while the adversarial and mismatch loss weight $(\gamma)$ was set at 0.9. The loss weights were adjusted incrementally according to the formula:

$$\begin{aligned} \begin{matrix} \alpha=\beta=\alpha_{0}\times r^{t} \\ \gamma=1-\alpha-\beta\end{matrix}\#\left( S1 \right) \end{aligned}$$

where $\alpha_{0}$ was set to 0.1 and $r$ to 1.002. This dynamic adjustment gradually shifts focus from adversarial robustness to spectral and structural accuracy as training progresses. The model uses a latent vector length of 800 and a clustering mechanism with $k=800$ to refine the training. The Adam optimizer’s beta parameters were set at $(0.9,0.999)$ to balance fast convergence with training stability, crucial for learning complex metasurface designs.

Section S6. Diverse near-field electric responses of metasurfaces with similar absorption spectra designed by AcGAN


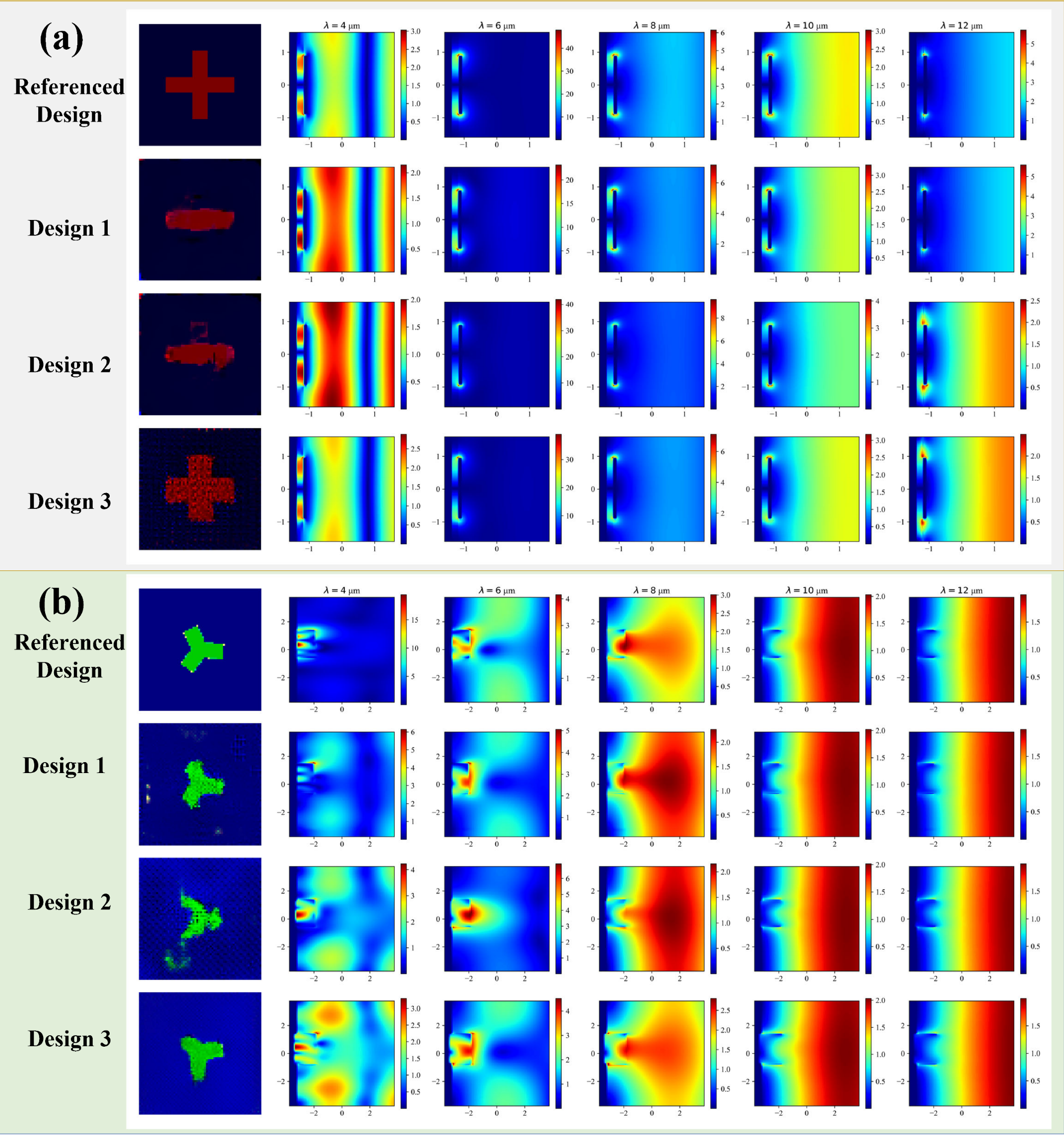


**Figure S3.** Near-field electric responses in the XZ plane for MIM and hybrid metasurface: (a) MIM metasurface: Showcases the near-field electric responses at various wavelengths (4 $\mu m$ to 12 $\mu m$) for MIM metasurface in **Figure 7**(f). The depth of the colors i reflects the magnitude of the near-field electric field strength. (b) Hybrid metasurface: Presents the near-field electric responses at wavelengths from 4 $\mu m$ to 12 $\mu m$ for hybrid metasurface in **Figure 7** (i).


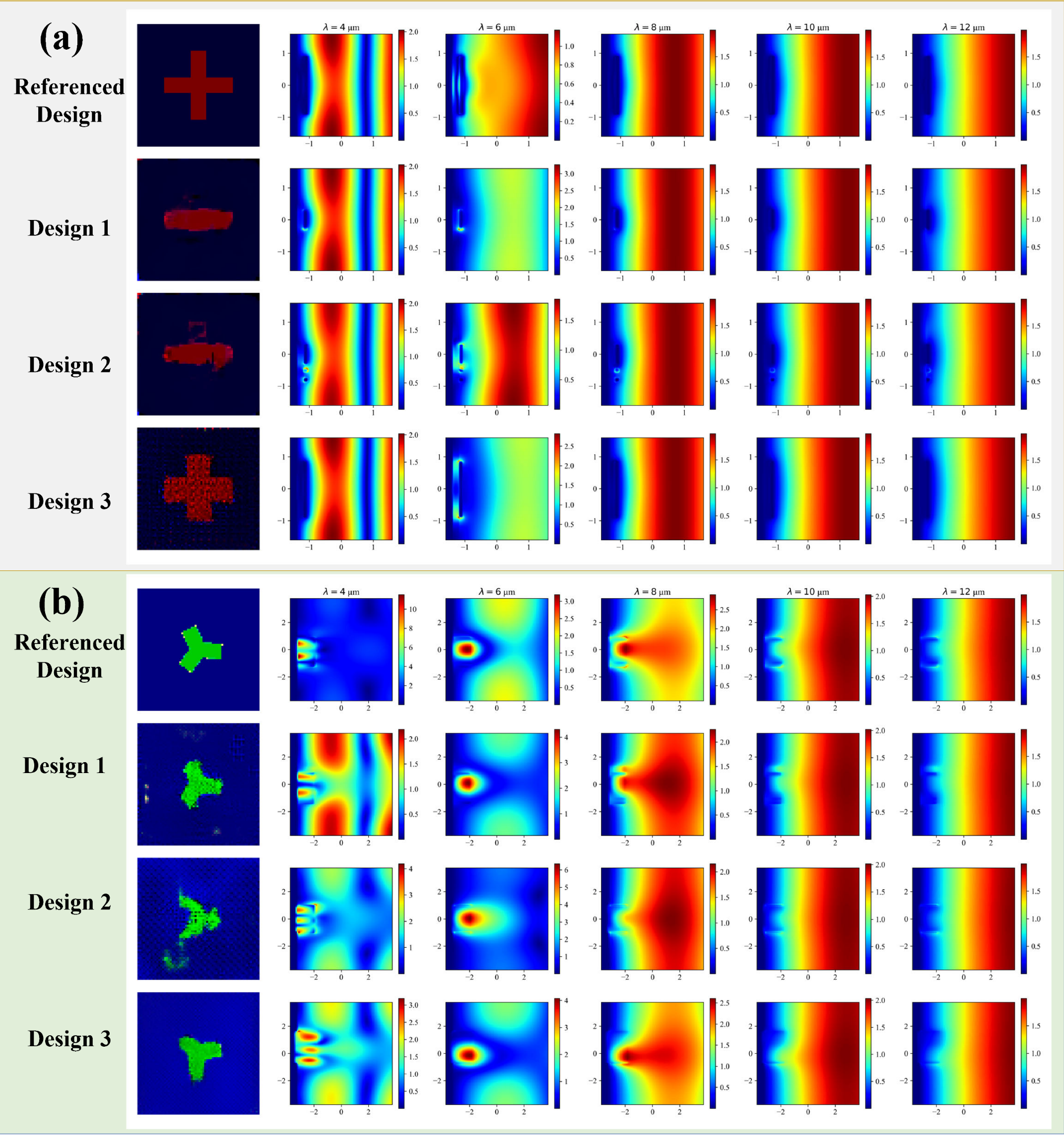


**Figure S4.** Near-field electric responses in the YZ plane for MIM and hybrid metasurface: (a) MIM metasurface: Showcases the near-field electric responses at various wavelengths (4 $\mu m$ to 12 $\mu m$) for MIM metasurface in **Figure 7** (f). The depth of the colors i reflects the magnitude of the near-field electric field strength. (b) Hybrid metasurface: Presents the near-field electric responses at wavelengths from 4 $\mu m$ to 12 $\mu m$ for hybrid metasurface in **Figure 7** (i).

The primary goal of our experiment is to evaluate the capability of AcGAN in designing metasurfaces that achieve both high electromagnetic fidelity and structural diversity, tailored to diverse electromagnetic applications. Specifically, we aim to demonstrate that AcGAN can generate metasurfaces with similar absorption spectra but varied near-field electric responses when analyzed across different spatial orientations (XY, XZ, and YZ planes). Due to space constraints, we have selected representative examples from the MIM and Hybrid structures.

These results reveal the magnitude of the electric fields across different spatial orientations (XY, XZ, and YZ planes), emphasizing regions of high and low EM activity. The images illustrate how the electric field varies across different planes, which is crucial for understanding energy distribution and the metasurface's effectiveness in manipulating EM waves. Each figure includes a color scale that represents the magnitude of the near-field electric response, with warmer colors indicating higher intensity and cooler colors representing lower intensity. The color bar quantifies the range of field intensities, allowing for a precise understanding of the field’s strength across different regions of the metasurface. Analysis of these figures shows that, despite having similar absorption spectra, the metasurfaces exhibit distinct near-field patterns across different orientations. This validates the hypothesis that AcGAN can introduce structural variability without compromising EM performance. This finding is significant as it highlights AcGAN’s potential in designing advanced optical devices where functional diversity is required alongside specific optical properties. This capability extends the utility of metasurfaces beyond conventional applications, enabling the creation of customized solutions for complex optical systems.

Section S7. Generation of four arbitrarily-defined spectra

To assess the AcGAN framework's capacity for designing metasurfaces corresponding to different spectral profiles, we synthesized four types of spectra, each crafted using specific mathematical expressions with defined parameter ranges. These spectra are discretized into 800 points, simulating measurements across a wavelength range from $4\mu m$ to $12\mu m$ :

1. **Gaussian Spectrum Generation**

Gaussian spectra are constructed as follows:

$$\begin{aligned} \boldsymbol{s}\left( \boldsymbol{i} \right)\mathbf{=}\boldsymbol{A}\mathbf{exp}\left( \mathbf{-}\frac{\left( \boldsymbol{i}\mathbf{-}\boldsymbol{\mu} \right)^{\boldsymbol{2}}}{\boldsymbol{2}\boldsymbol{\sigma}^{\boldsymbol{2}}} \right)\boldsymbol{\#}\left( \mathbf{S2} \right) \end{aligned}$$

where $i$ indexes the discretized points. The mean $\mu$ varies randomly within $\left[ -2,2 \right]$, the standard deviation $\sigma$ within $\left[ 0.5,2 \right]$, and the amplitude $A$ is scaled between $\left[ 0.5,1.0 \right]$ to normalize the peak amplitude.

**2. Gate-shaped Spectrum Generation**

Gate-shaped spectra are defined by a constant amplitude over a random interval:

$$\begin{aligned} s\left( i \right)=\left\{ \begin{matrix} A & \text{if }l\leq i\leq u \\ 0 & \text{otherwise } \end{matrix} \right.\#\left( S3 \right) \end{aligned}$$

The boundaries $l$ and $u$ of the active interval are selected such that the width is between 50 and 200 points, and the amplitude $A$ ranges from 0.6 to 1.0 .

**3. Lorentzian Spectrum Generation**

Lorentzian spectra are described by:

$$\begin{aligned} \boldsymbol{s}\left( \boldsymbol{i} \right)\mathbf{=}\frac{\boldsymbol{A\gamma}}{\boldsymbol{\pi}\left( \left( \boldsymbol{i}\mathbf{-}\boldsymbol{x}_{\boldsymbol{0}} \right)^{\boldsymbol{2}}\mathbf{+}\boldsymbol{\gamma}^{\boldsymbol{2}} \right)}\boldsymbol{\#}\left( \mathbf{S4} \right) \end{aligned}$$

The parameter $x_{0}$, the center of the peak, is randomly chosen from $\left[ -5,5 \right],\gamma$, indicating the width, varies between $\left[ 0.1,2 \right]$, and the amplitude $A$ is adjusted to fall within $\left[ 0.6,1.0 \right]$.

**4. Fano Resonance Spectrum Generation**

Fano resonance profiles are generated using:

$$\begin{aligned} \boldsymbol{s}\left( \boldsymbol{i} \right)\mathbf{=}\boldsymbol{A}\frac{\left( \boldsymbol{q\gamma}\mathbf{+}\boldsymbol{i}\mathbf{-}\boldsymbol{x}_{\boldsymbol{0}} \right)^{\boldsymbol{2}}}{\boldsymbol{\gamma}^{\boldsymbol{2}}\mathbf{+}\left( \boldsymbol{i}\mathbf{-}\boldsymbol{x}_{\boldsymbol{0}} \right)^{\boldsymbol{2}}}\boldsymbol{\#}\left( \mathbf{S5} \right) \end{aligned}$$

where $q$ represents the asymmetry parameter with a range between 3 and 7 . Other parameters, $x_{0}$ and $\gamma$, are set similarly to the Lorentzian spectrum, with amplitude $A$ being normalized similarly.

These synthesized spectra, mapped across 800 data points, serve as test cases for evaluating AcGAN's design capabilities under varied spectral conditions. This setup mimics realistic scenarios by capturing diverse spectral behaviors within the specified wavelength range, ensuring a robust evaluation of the framework's performance in generating metasurface designs tailored to specific electromagnetic properties.

Section S8. The performance of AcGAN of arbitrarily-defined spectrum

**
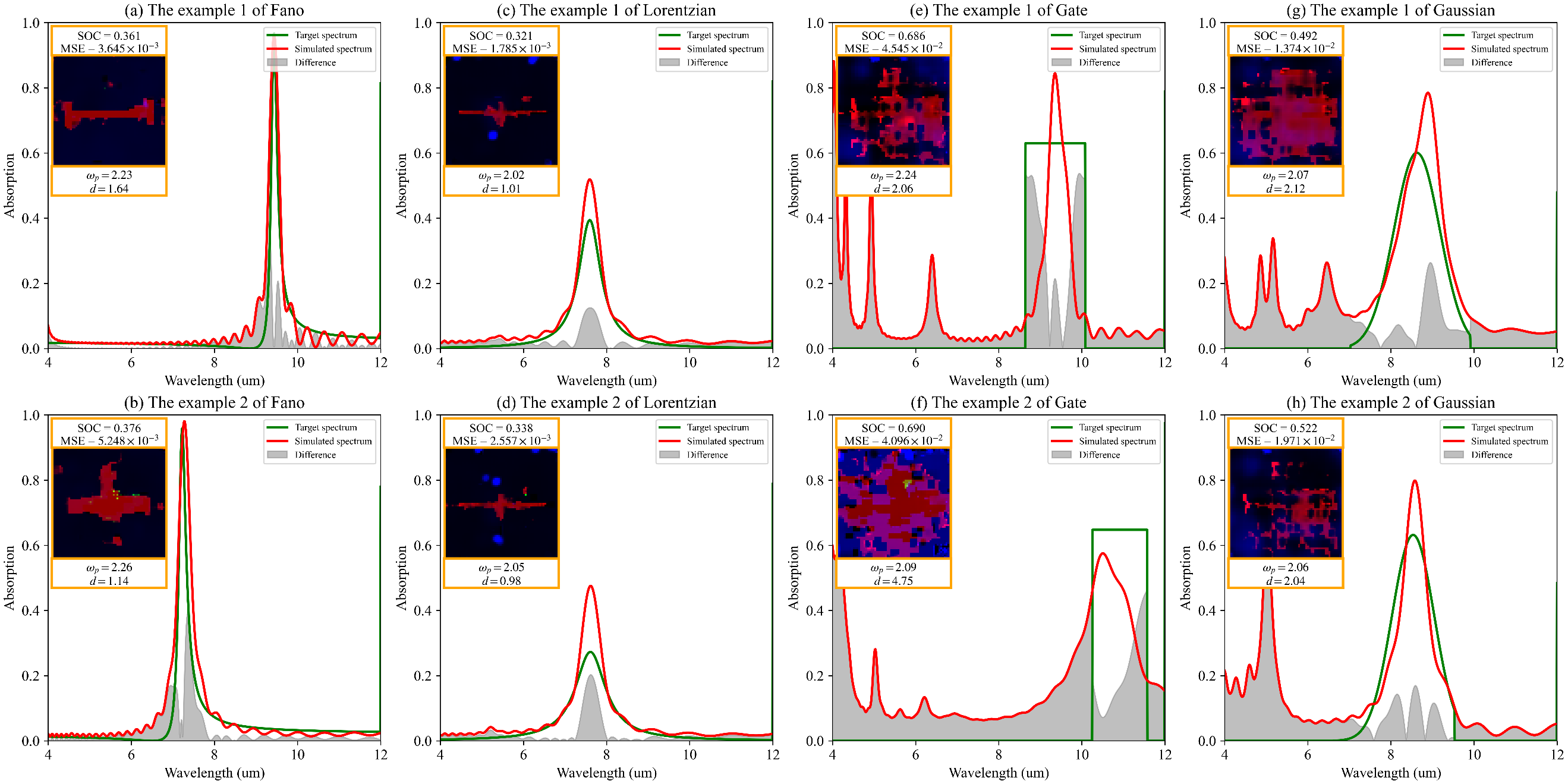
**

**Figure S5.** AcGAN response to to different spectral types for which there are no corresponding structures in the dataset. For each type, two examples are shown to demonstrate AcGAN's predictive accuracy compared to the target spectra. The simulated spectra (red curves) are compared with the target spectra (green curves) to highlight the model's precision. Each subplot includes SOC and MSE metrics to quantify the discrepancy between the simulated and target spectra. Gray shaded areas indicate significant spectral mismatches. Insets within each panel display the corresponding metasurface structures, illustrating the correlation between spectral properties and physical configurations.

Section S9. Correlation between weighted distance and SOC for various spectral types


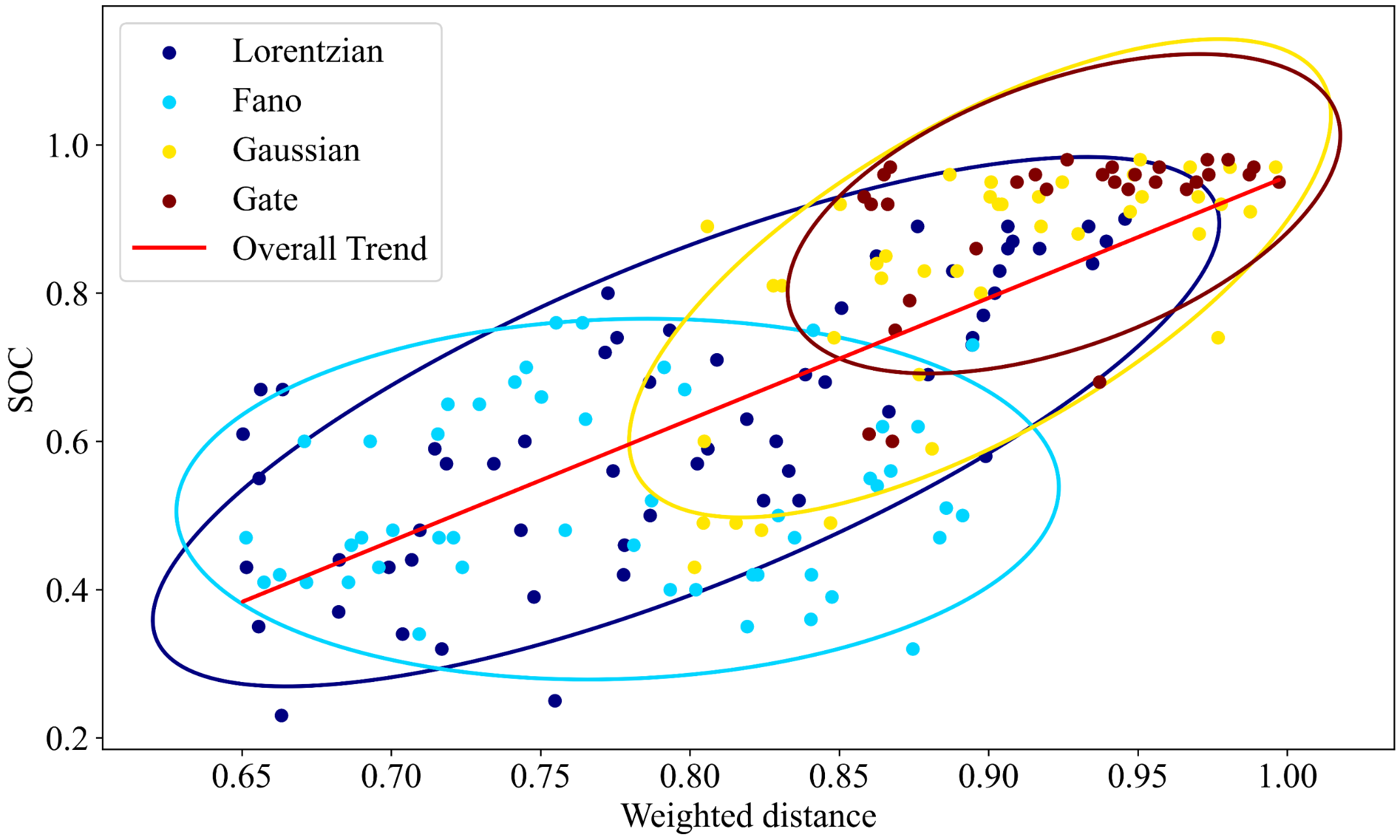


**Figure S6.** Correlation between weighted distance and SOC for various spectral types. The scatter plot shows the relationship between the weighted distance from dataset cluster centers and the SOC for generated spectra. Points represent Gaussian (blue), Gate (yellow), Lorentzian (black), and Fano (red) types. Ellipses represent the data distribution for each type. The overall trend (red line) indicates that closer proximity to training data (lower weighted distance) generally corresponds to higher SOC, indicating worse model performance.

Section S10. Ablation study

**Table S4.** Ablation study comparing AcGAN performances with and without cluster controller

| **Turn on the Cluster Controller** | **Average MSE** | **Average SOC** |
| --- | --- | --- |
| False | $9.770\times{10}^{-3}$ | 0.434 |
| **True** | $\boldsymbol{1.120\times}\boldsymbol{10}^{\boldsymbol{-3}}$ | **0.139** |

The inclusion of the cluster controller significantly enhances model accuracy. With the cluster controller activated, the average MSE decreased from $9.770\times{10}^{-3}$ to $1.120\times{10}^{-3}$, and the SOC improved from 0.434 to 0.139 (Table S4). This stark improvement underscores the controller’s ability to refine the spectral dataset’s representation, which aids in generating metasurfaces that more closely match the desired spectral characteristics. Since disabling the cluster controller is equivalent to removing the vector $v$, these results explicitly demonstrate the critical role of the vector $v$, in providing global spectral context. It suggests that the cluster controller effectively captures pivotal spectral features that are essential for the network to generate high-fidelity designs.

**Table S5.** Ablation study comparing AcGAN performances with and without AnchorNet integration in generator and discriminator

| **Turn on the AnchorNet on the generator** | **Turn on the AnchorNet on the discriminator** | **Average MSE** | **Average SOC** |
| --- | --- | --- | --- |
| False | False | $2.674\times{10}^{-3}$ | 0.228 |
| True | False | $2.742\times{10}^{-3}$ | 0.224 |
| False | True | $2.512\times{10}^{-3}$ | 0.220 |
| **True** | **True** | $\boldsymbol{1.120\times}\boldsymbol{10}^{\boldsymbol{-3}}$ | **0.139** |

Incorporating AnchorNet within both the generator and the discriminator significantly optimizes the network’s performance. The configuration where AnchorNet is active in both the generator and discriminator simultaneously yielded the best results, with an MSE of $1.120\times{10}^{-3}$ and an SOC of 0.139 (Table S5). This dual deployment enables a more cohesive and accurate evaluation of generated metasurfaces against the target spectra, demonstrating that synchronized feedback between these components is crucial for enhancing the fidelity of generated designs.

**Table S6.** Impact of $\xi$-value on AcGAN performance

| $\boldsymbol{\xi}$ **value** | **Average MSE** | **Average SOC** |
| --- | --- | --- |
| 1 | $2.613\times{10}^{-3}$ | 0.207 |
| 2 | $\boldsymbol{1.120\times}\boldsymbol{10}^{\boldsymbol{-3}}$ | **0.139** |
| 4 | $3.522\times{10}^{-3}$ | 0.221 |
| 8 | $9.698\times{10}^{-3}$ | 0.415 |

The balance between discriminator and generator training frequencies, controlled by the $\xi$-value, plays a crucial role in stabilizing the adversarial training process. We varied the $\xi$ -value to adjust how many times the generator updates for each discriminator update. Our findings indicated that a $\xi$ -value of 2 provided the best performance, minimizing MSE to $1.120\times{10}^{-3}$ and SOC to 0.139, suggesting that too frequent generator updates relative to the discriminator might lead to inefficiencies, potentially due to overfitting or unstable adversarial dynamics.

**Table S7.** Effect of initial spectral loss weight 𝛼 on AcGAN performance

| Initial spectral loss weights $\alpha$ | **Average MSE** | **Average SOC** |
| --- | --- | --- |
| 0.1 | $\boldsymbol{1.120\times}\boldsymbol{10}^{\boldsymbol{-3}}$ | **0.139** |
| 0.2 | $3.003\times{10}^{-3}$ | 0.271 |
| 0.3 | $3.451\times{10}^{-3}$ | 0.309 |
| 0.4 | $3.719\times{10}^{-3}$ | 0.333 |
| 0.5 | $5.405\times{10}^{-3}$ | 0.384 |
| 0.6 | $8.830\times{10}^{-3}$ | 0.466 |
| 0.7 | $1.075\times{10}^{-2}$ | 0.486 |
| 0.8 | $1.023\times{10}^{-2}$ | 0.488 |
| 0.9 | $9.705\times{10}^{-3}$ | 0.475 |
| 1.0 | $1.457\times{10}^{-2}$ | 0.550 |
| 2.0 | $1.297\times{10}^{-2}$ | 0.530 |

The weight of the spectral loss, managed by α, directly impacts how the feedback from the AnchorNet shapes the generator’s outputs. Surprisingly, an α of 0.1 yielded the most favorable outcomes, with the lowest MSE and highest SOC, indicating a well-balanced adversarial and spectral loss contribution. This suggests that while spectral alignment is critical, its dominance in the loss function must be carefully calibrated to avoid overshadowing the adversarial component necessary for diverse and generalizable metasurface design.

**Table S8.** AcGAN performance across various latent sizes

| **Latent size** | **Average MSE** | **Average SOC** |
| --- | --- | --- |
| 100 | $3.116\times{10}^{-3}$ | 0.238 |
| 200 | $3.388\times{10}^{-3}$ | 0.280 |
| 400 | $3.153\times{10}^{-3}$ | 0.236 |
| 800 | $\boldsymbol{1.120\times}\boldsymbol{10}^{\boldsymbol{-3}}$ | **0.139** |
| 1600 | $1.403\times{10}^{-2}$ | 0.529 |

Exploring different sizes of the latent space, we assessed how the dimensionality influences the model's ability to encapsulate and generate diverse metasurface designs. A latent size of 800 was found to be optimal, striking a balance between complexity and manageability, and significantly outperforming smaller and larger sizes in both MSE and SOC. This highlights the importance of an appropriately sized latent space in capturing the necessary variability in metasurface designs without introducing excessive noise or complexity.

**Table S9.** AcGAN performance variation with different batch sizes

| **Batch size** | **Average MSE** | **Average SOC** |
| --- | --- | --- |
| 64 | $3.102\times{10}^{-3}$ | 0.223 |
| 128 | $3.318\times{10}^{-3}$ | 0.234 |
| 256 | $\boldsymbol{1.120\times}\boldsymbol{10}^{\boldsymbol{-3}}$ | **0.139** |
| 512 | $2.730\times{10}^{-3}$ | 0.213 |

Our analysis extended to the effect of batch size on model performance. Smaller batch sizes generally led to higher MSE and lower SOC, underscoring the benefits of larger batch sizes in stabilizing training updates and gradient estimations. A batch size of 256 demonstrated the best performance, optimizing both MSE and SOC, facilitating more stable and reliable training cycles.

**Table S10.** Impact of $k$-value on AcGAN performance

| $k$ value | **Average MSE** | **Average SOC** |
| --- | --- | --- |
| 100 | $6.993\times{10}^{-3}$ | 0.313 |
| 200 | $3.388\times{10}^{-3}$ | 0.261 |
| 400 | $3.013\times{10}^{-3}$ | 0.216 |
| 800 | $\boldsymbol{1.120\times}\boldsymbol{10}^{\boldsymbol{-3}}$ | **0.139** |
| 1600 | $3.085\times{10}^{-3}$ | 0.379 |

To assess the influence of the number of spectral clusters on the controller’s effectiveness, we varied the value of $k$ and measured the resulting design fidelity. As shown in Table S10, performance improved steadily with larger $k$ values up to $k=800$, yielding the lowest MSE and SOC. However, setting $k=1600$ led to performance degradation, likely due to over-fragmentation of the spectral space and reduced generalization. These results highlight that $k=800$ offers the best trade-off between spectral discrimination and robustness.
